# Parameter set for computer-assisted texture analysis of fetal brain

**DOI:** 10.1186/s13104-016-2300-3

**Published:** 2016-11-25

**Authors:** Hugues Gentillon, Ludomir Stefańczyk, Michał Strzelecki, Maria Respondek-Liberska

**Affiliations:** 1 Department of Radiology and Diagnostic Imaging, Barlicki University Hospital, Medical University of Lodz, Lodz, Poland; 2 Institute of Electronics, The Faculty of Electrical, Electronic, Computer and Control Engineering, Technical University of Lodz, Lodz, Poland; 3 Diagnosis and Prevention of Congenital Malformations, Instytut Centrum Zdrowia Matki Polki, Lodz, Poland

**Keywords:** Histogram, Wavelets, Computer-assisted radiology, Hugues Gentillon, Teleradiology, Prenatal development, Fetal brain, Mazda, b11, Medical cybernetics, Artificial intelligence, Computational visual cognition

## Abstract

**Background:**

Magnetic resonance data were collected from a diverse population of gravid women to objectively compare the quality of 1.5-tesla (1.5 T) versus 3-T magnetic resonance imaging of the developing human brain. MaZda and B11 computational-visual cognition tools were used to process 2D images. We proposed a wavelet-based parameter and two novel histogram-based parameters for Fisher texture analysis in three-dimensional space.

**Results:**

Wavenhl, focus index, and dispersion index revealed better quality for 3 T. Though both 1.5 and 3 T images were 16-bit DICOM encoded, nearly 16 and 12 usable bits were measured in 3 and 1.5 T images, respectively. The four-bit padding observed in 1.5 T K-space encoding mimics noise by adding illusionistic details, which are not really part of the image. In contrast, zero-bit padding in 3 T provides space for storing more details and increases the likelihood of noise but as well as edges, which in turn are very crucial for differentiation of closely related anatomical structures.

**Conclusions:**

Both encoding modes are possible with both units, but higher 3 T resolution is the main difference. It contributes to higher perceived and available dynamic range. Apart from surprisingly larger Fisher coefficient, no significant difference was observed when testing was conducted with down-converted 8-bit BMP images.

**Electronic supplementary material:**

The online version of this article (doi:10.1186/s13104-016-2300-3) contains supplementary material, which is available to authorized users.

## Background

**Fig. 1:**
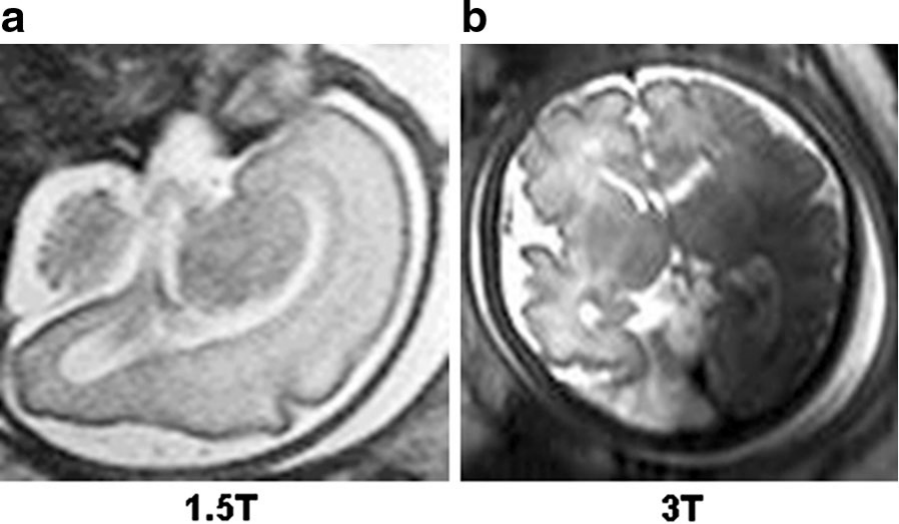
“Do you see what I see?”. Fetal brain. **a** *Left* 1.5 T. **b** *Right* 3 T. This figure was incorporated and so-titled in this manuscript to illustrate that magnetic resonance (MR) images can be used for evaluation of achromatic vision and sensitivity to change in *grayscale* quality between different subjects. For fair comparison, this exercise should be blinded. It is recommended to have at least two radiologists, if not available, medically-trained practitioners or any volunteers (blinded) and one examiner (also blinded) to look at the images side-by-side, on two medical-diagnostic monitors, engineered for 16-bit display (same brand/model in natively flat display mode: i.e. without any added enhancements). All in-computer/in-monitor RT (real-time) editing features must be turned off (incl. hardware/software rendering): n.b. 16-bit of true data has more room for “contrast booster”—which is essentially an illusion, as a result of post-editing artifacts, not really part of the image. In this investigation, volunteers were asked to identify the images unlabeled (, of course). It was observed that both images were equally sharp (in pass-through mode), for a normal, naked human eye. There flows the explanation for the research goal to mathematically determine which MR modality actually produces images with more captured details. Both images were 16-bit encoded, and the difference could not be measured via “perceived dynamic range” (visible details). With computer vision software, 1.5/3 T images can be numerically decoded to accurately assess “available dynamic range” (visible/invisible details)

Computer is a non-biological copy of the human brain. As its use increases day-by-day in medicine, various transdisciplinary approaches emerge. Among them is texture analysis, the evolving cybernetics of radiology. This experiment is a translational study seeking to objectively compare the quality of 1.5-tesla (1.5 T) versus 3-T magnetic resonance imaging (MRI) of the developing human brain (Fig. 1), in order to determine whether the extra administrative cost is worthy for the patient and the healthcare system. First there was a need to develop an objective methodology to collect and process the data and subsequently justify its necessity and dispel distrust of financial burden.

### MRI replacing USG during pregnancy examination

**Fig. 2:**
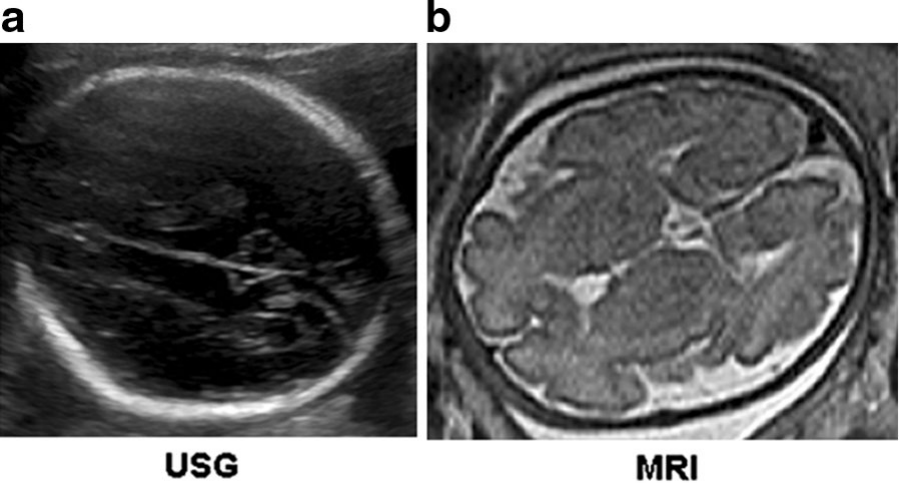
Ultrasonography (USG) vs. magnetic resonance imaging (MRI). Fetal MRI was used as a complimentary modality to USG. **a** USG. **b** MRI

In terms of safety, ultrasonography (USG) remains the gold standard for prenatal central nervous system (CNS) imaging [1]. It is used for prevention and diagnosis of congenital malformation [2, 3]. Nevertheless, there are cases which benefit from alternative imaging techniques like 1.5 or 3 T MRI [4–9]. Besides spotting maternal abnormalities, supplemental information from magnetic resonance imaging is also crucial for identification of fetal anatomy and pathology [10, 11]. MRI is also being increasingly used as correlative imaging modality in pregnancy—because it uses no ionizing radiation, has no known teratogenic effects, provides excellent soft-tissue contrast, and has multiple planes for reconstruction and large field of view, allowing better depiction of neuroanatomy in fetuses with large or complex anomalies [12–15]. Compared to magnetic resonance imaging, USG is more affordable and widely used in many countries, as a radiologic tool in routine examination of pregnant women, especially in the detection of fetal anomalies, at about 20th week of gestation [2, 3]. On the other hand, 1.5 and 3 T MRI scanners produce superior CNS images (Fig. 2) but are not cost-effectively built. These units are massive and expensive [16–20]. They are mostly available in big-budget hospitals and wealthy medical research centers. Because of such disadvantages, carrying research with MRI is more likely to be impeded. As a result, little information is known about its relevance to potential beneficiaries. MRI is bureaucratically recommended only if it is essential in some health care systems administrated by a central office (e.g. Sweden) [21]. Physicians are even liable for part of MRI examination cost in some territories (e.g. Poland) [22, 23]. Thus relevance to beneficiaries is likely to be affected in such health care systems.

### What is texture analysis?

Texture analysis is an artificial process involving quantification of image quality by means of parametric features to characterize regions of interest (ROI) [24–26]. While texture analysis is still far from replacing the clinician eye, some of its features warrant further consideration for integration into medical practice. To this date, texture analysis is used in pre-diagnosis of the globally pandemic disease of tuberculosis [27–29]. In Bangladesh, for example, radiologists and radio-technicians quickly screen patients displaying tuberculosis (TB) signs with CAD4TB texture analysis software [30–33]. This medical application of texture analysis is sponsored by The World Health Organization, at a mere cost of $3 per CAD4TB test. Only patients pre-diagnosed TB-positive by CAD4TB software receive the more expensive molecular examination known as GeneXpert TB Diagnosis and Resistance Test [30–33]. In the aforementioned example, an increase in the rate of early- detection of TB has been reported in clinics where texture analysis is used as a pre-diagnostic tool [30–33]. Apart from image encoding statistics, other factors also affect how clinicians perceive medical image appearance: e.g. acutance or subjective quality factor, human eye’s contrast sensitivity function, modulation transfer function or spatial frequency response, image displayed height, viewing distance, positioning of the subjects (stand, seat), radiology LCD (liquid crystal display) screen characteristics (anti-glare, glossy, size, resolution, calibration), etc [34–36].

### Wavelets-based texture analysis

Wavelets are texture analysis parameters which classify intrinsic properties of an image into matrix layers, and therefore they are very powerful tools for differentiating and matching patterns in regions of interest. Wavelets are used, for example, in forensic investigation to match fingerprints [37], in diagnostic radiology to detect suspected diseases [38], and in many other applications where digital imaging is needed [37].

### Histogram-based texture analysis

**Fig. 3:**
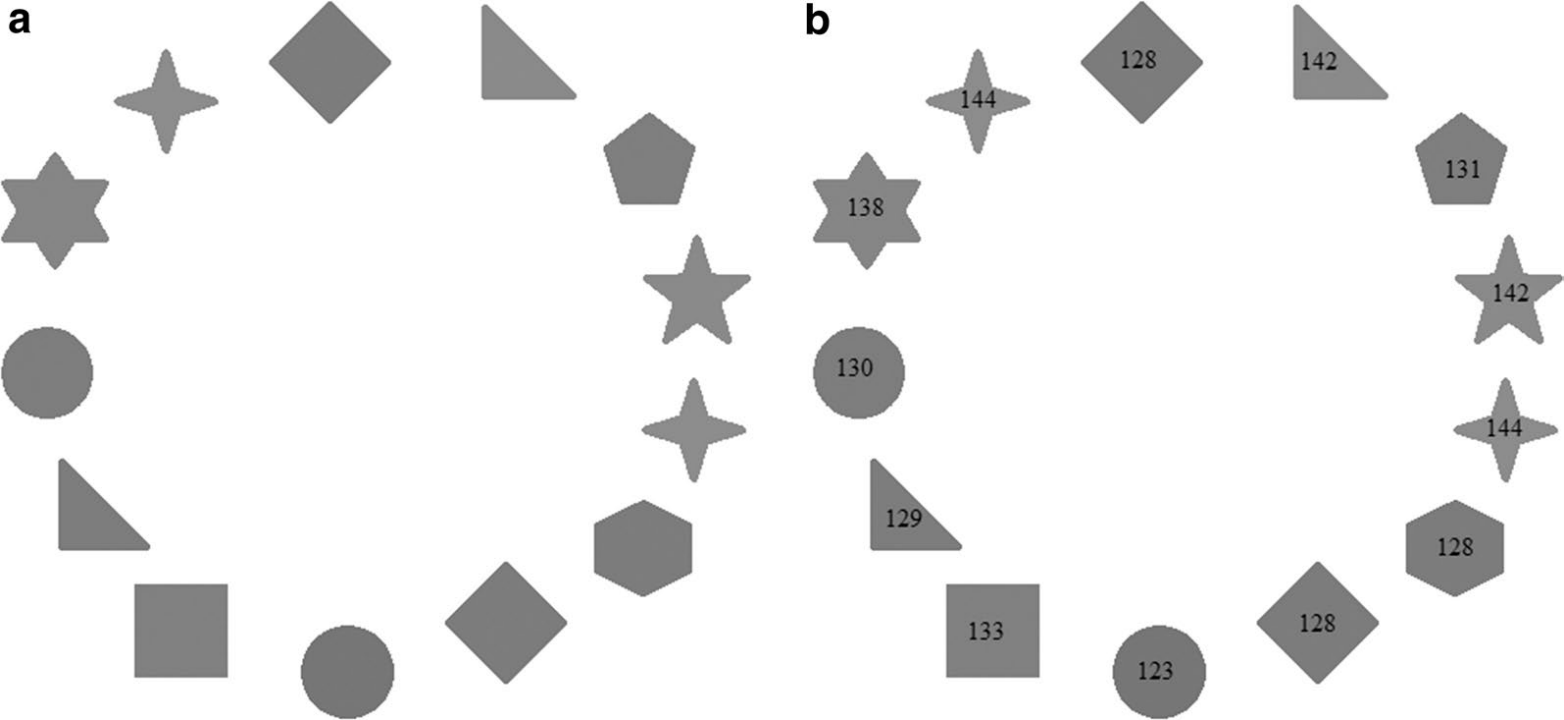
Varying shades of *grey*. **a** Arrange from lighter to darker? **b** Answers to Puzzle as per measurements from computer vision software. Again, test subjects should be blinded

To fully grasp the concept behind the methodology used in this experiment, it is important to first comprehend or at least have a minimum knowledge about the purpose, functionality, and usefulness of histogram in digital imaging. Just as the aforementioned brain images in Fig. 1, the quality and texture of every shape in Fig. 3 may not be interpreted in the same way by different pairs of human eyes.

**Fig. 4:**
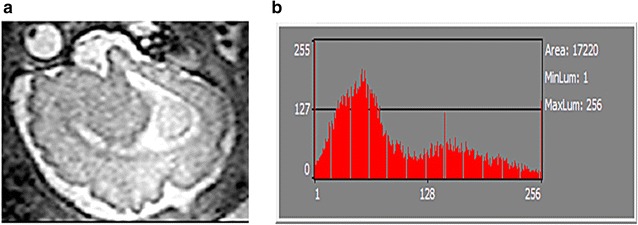
Histogram representation of fetal brain. **a** MRI of fetal brain. **b** Histogram of the whole image

**Fig. 5:**
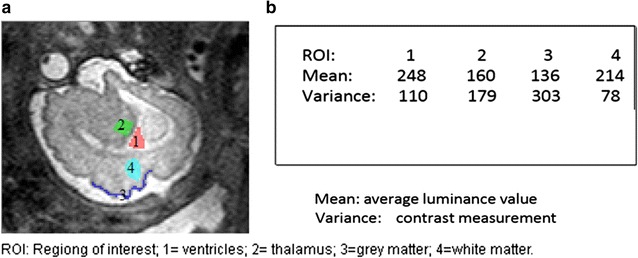
Regions of interest. **a** *ROI 1* ventricles; *2* thalamus; *3* grey matter; *4* white matter. **b** Histogram parameters from texture analysis with MaZda

**Fig. 6:**
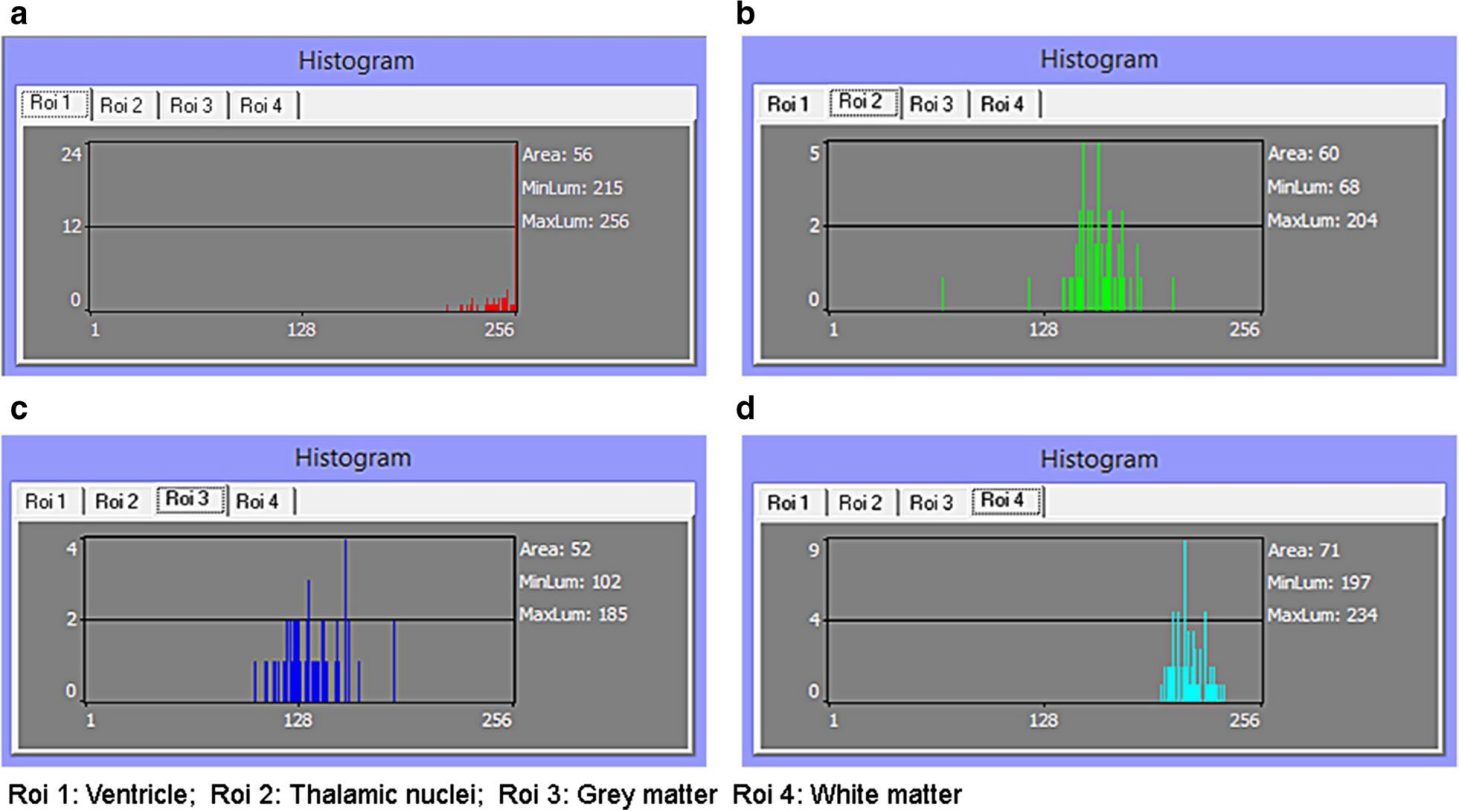
ROI analysis. **a** *ROI 1* Ventricles; **b** *ROI 2* Thalamic nuclei; **c** *ROI 3* Grey matter; **d** *ROI 4* White matter. There exist several techniques and methods of texture analysis. Non-parametric graphs (e.g. histogram, box plot) would be indeed a simple alternative to conduct this study. As shown in the figures, MaZda does display histogram of 8-bit but not for 16-bit DICOM image. Also, there are issues with drawing conclusions straight from histograms and boxplots. They are pictorial representations and thus are indirect methods. Furthermore non-parametric interpretation may not be as precise and accurate as parametric quantification. Therefore, parametric quantification was used to assess image quality rather than conventional appearance. 3D, non-parametric graphs are also possible with collateral usage of MaZda (version 5) and B11 (version 3.3) in training mode. The problem with training methods is that errors might occur as a result of overtraining the network. Hence, raw analysis was performed

Histogram parameters are digital imaging tools which analyze textural characteristics of a picture by measuring their quality [39, 40]. Such parameters can differentiate texture by quantifying, for example, resolution, sharpness, noise, actual edge details and artifacts, negative and positive exposures, as well as aliasing and moiré—i.e. superimposed patterns in MR images [41–44]; moiré effect is most commonly observed in uncontrolled TV interviews recorded with older cameras—and the interviewees were wearing pinstriped clothing [45, 46]. In some software, histogram is customized for 8-bit graphical visualization to fit the display of conventional RGB monitors, with luminance value of 0–255 (8 bits = 28 = 256). In other words, a 16-bit image would be down-sample in order to display its geometric representation on such computational histograms (Figs. 4, 5, 6). A Histogram display in MaZda, on the other hand, does not graphically display pixel values larger than 255. However, it is able to quantify 16-bit DICOM (Digital Imaging and Communications in Medicine), evaluate quality of different MR modalities, and differentiate ROIs. *Is this limit a histogramatic error?* No, it is not. A radiology monitor of, probably, the size of a theatre screen is needed to visually display a linear representation of every value in a 16-bit image (2^16^ − 1 = 65,535). In spite of the graphical limitations, histogram-based texture analysis is more efficient than edge detection for pixel segmentation and identification of region boundaries [47, 48]. Some histogram parameters (e.g. mean, variance, kurtosis, skewness) can be used to differentiate noise and related artifacts from very sharp details such as edges (region boundaries).

## Methods

This multidisciplinary research was approved by a relevant bioethics committee, consisting of emeritus professors and senior researchers from the Medical University of Lodz (MUL), Barlicki University Hospitals (BUH), Polish Mother’s Memorial Hospital Research Institute (ICZMP) et al. Written informed consent was obtained from all subjects, and the methods were carried out in accordance with the approved guidelines (see end of the manuscript for registration number). The experiment was divided into three parts.

### Research tools

Firstly, some quality-control checks were performed before designing this research. Images (Fig. 1) were imported and uncompressed in Photoshop CS6 64-bit Extended. The software was set to HDR (high-bit-depth resolution) mode (i.e. floating-point numeric representation: up to 32 bits × 3 channels). RAW-formatted images were simultaneously examined with radiology computer systems (i.e. high-end hardware/accessories, monitors capable to render 16-bit grayscale natively; memory-intensive controller/graphic card, Phillips DICOM Viewer R3.0 SP3, and LCD calibration software). With proper-matching adjustments, difference between the two images were imperceptibly unnoticeable. That was a conundrum which proved the need to carry out quality-assessment research with MaZda and B11.

### Trials

BUH (1.5 T unit) and ICZMP (3 T unit) radiology departments prescribed MRI to patients for further differential-diagnostic investigation—rather than just a concomitant adjunct to ultrasound. 288 MR images of normal fetal brain were used in the main assessment and 72 MR images of normal fetal brain in the supplementary trials (see Table 1 for further description).

**Table 1:** Sample description

	1.5 T	3 T	Total
A. Main trials	144	144	288
B. Supplementary trials	36	36	72
Group 1	20	20	40
Group 2	16	16	32

A. *Main trials* 288 MR images, categorized by magnetic field strength of scanner (144 × 1.5 and 144 × 3 T). B. *Supplementary trials* 72 MR images (36 × 1.5 and 36 × 3 T). Samples used in the supplementary trials were further divided into two categories, by gestational age: *group 1* 20–28 weeks; *group 2* 29–40 weeks

MR images came from MRI studies of different patients who had fetal MRI as a complimentary modality to ultrasonography and echocardiography, to further assess and confirm the diagnosis of suspected anomalies affecting the fetuses as well as the pregnant patients. Most participants were seeking remedies for conditions not related to fetal brain. Research hospitals in Poland are among the forefront pioneers in prenatal diagnosis of congenital malformation, in utero surgery, cardiology and minimally-invasive cardio-surgery [49]. In spite of its stigmatic plight, modern cardiology arguably began in Lodz, Poland, with advances in medical electronics about 70 years ago [50]. This background history explained why patients travelled there to seek second opinion rather than just for the known association of cost-effective treatment in Poland. The selected samples were diverse—i.e. subjects were not closely related (not monozygotic twins, not dizygotic twins, not consanguineous twins, etc). Gestational ages were between 20 and 40 weeks. Patients underwent either 1.5 T scan at BUH or 3 T scan at ICZMP—but not both. It was deemed irrelevant in terms of assessment of image quality as well as for good patient care and fetal rights (i.e. moral and legal rights of human fetuses) [51]. In recent years, physical appearances of subjects (such as eye shapes) have been documented to cause some photography cameras to capture deceptive images in auto-mode—a dysfunction due to faulty-engineering and bug compatibility [52–54]. To our knowledge, such phenomena have not been reported in radiological imaging. Furthermore it is the responsibility of manufacturers to ensure that MR units are capable to capture good quality sequences, regardless of mother and/or fetus phenotypes. In this investigation, the artifacts observed in excluded samples were consequences of fetal movement and inadequate settings by radiographers, not due to phenotypic characteristics. Beside consent-and-publication agreement, the objective of this research did not rationally necessitate broadcasting further descriptions about patients’ background/identities (incl. phenotypes and anamneses) [51]. Therefore, such details were omitted in this manuscript as well as in the datasets [51].

**Fig. 7:**
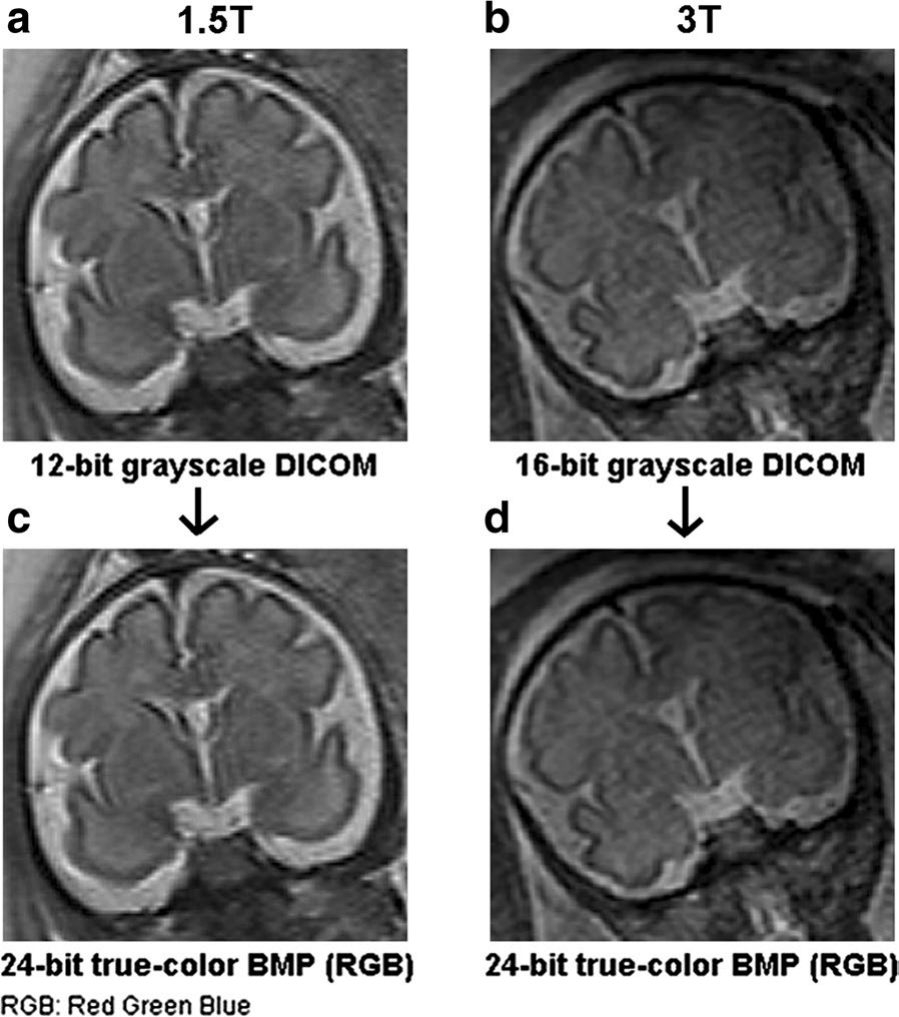
**a** 1.5 T MR image of a fetal brain: 12-bit DICOM format, coronal section. **b** 3 T MR image of a different subject: 16-bit DICOM, coronal plane. **c** Same image shown in “**a**” after conversion to BMP. **d** Same image shown in “**b**” after conversion to BMP

**Fig. 8:**
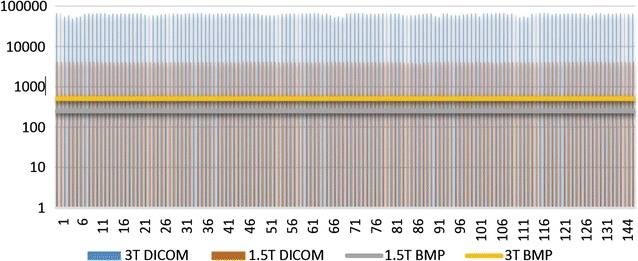
Measured usable bits (pixel values). 3/1.5 T BMP ≈ 256; 1.5 T DICOM ≈ 4096; 3 T DICOM ≈ 65,536

MaZda software package 5 (B11 included) was used for quantification of MR images. A wavelet-based parameter (wavenhl) was combined with two novel histogram-based parameters (focus index, dispersion index) to perform Fisher-texture analysis in three-dimensional space. It was hypothesized that Wavenhl could fingerprint (match) regions of interest—i.e. as per human anatomy, the building blocks of normal thalamus, for example, is the same regardless of subjects and MR modalities. Two parameters which were not sensitive to minute variations in phenotypes were favored in this research. Hence, focus index and dispersion index were utilized for assessing image quality in terms of resolution, sharpness, aliasing and moiré, noise, actual edge details and artifacts. MicroDicom was used to extract images from teleradiology network systems and storage media containing raw data of 3 and 1.5 T MRI studies. One DICOM and one BMP (also known as Bitmap: Microsoft Windows Device-Independent Bitmap file) were generated from each MR sequence to create four batches of images: 3 T DICOM, 1.5 T DICOM, 3 T BMP, and 1.5 T BMP. All files were extracted in their native size and resolution. Both 3 and 1.5 T sequences were processed as Little-Endian, Implicit and 16-bit uncompressed DICOM (RAW). All BMP images were encoded with no down- sampling of resolution. Both 3/1.5 T sequences were simultaneously exported as 16-bit DICOM, down-converted to 8-bit BMP (color space: RGB-24: 8 bits × 3 channels), and batch-processed. Note that MicroDicom applied equal, least-significant-bit (LSB) degradation to both 3/1.5 T BMP images. The quality and the information loss in both 3/1.5 T images were assessed with MaZda, a texture analysis software which measures image statistics in various file formats [4, 5, 55]. Histogram is the only feature in MaZda version 5, to numerically measure available dynamic range in 16-bit images. Histogram parameters were then used to calculate focus index values (skewness-to-mean kurtosis ratio) and dispersion index values (variance-to-mean ratio), which in turn serve as tools to evaluate the retention of stored, tonal details in BMP ROIs—compared to the same ROIs in the original DICOM file (Figs. 4, 5, 6, 7, 8; Additional file 1: Dataset 1). MaZda measured nearly 16 and 12 usable bits in 3 and 1.5-T DICOM images, respectively (Fig. 8; Additional file 1: Dataset 1). No significant difference was observed when testing was conducted with degraded BMP images (max. 8 bits in both) (Fig. 8; Additional file 1: Dataset 1).

Usable bit (stored bit) was used as a parametric factor rather than allocated or high bit, as the primary research interest was a quest to assess quality in terms of actual, captured details—free from illusionistic embellishments. In other words, a DICOM file may appear as or show properties display of a 16-bit image on a computer; when in reality it only holds 12 bits of the real thing. Many of the measured 16-bit DICOM images contain much less than 65,536 tonal patterns per pixel. It is due to the fact that not all allocated bits contain real captured details but bogus data. Reported usable bit can be simply obtained with any software which has a DICOM header viewer or editor. In this experiment, reported usable bits (in the DICOM headers) were first checked with MicroDicom. Then these metadata were double-checked and subsequently measured with MaZda to determine actual stored bit in every image. Measured usable bit does not always correspond to reported usable bit. Such discrepancies resulted from rounding errors in MR units. Thus the choice to go with measured usable bit was factually validated (Fig. 8; Additional file 1: Dataset 1). Images with 4–5 mm thickness and highest measured usable bits were selected. For texture segmentation, coronal plane was ideal because more images could be obtained from sequences with all four visible ROIs (i.e. thalamus, ventricles, grey matter and white matter). Furthermore it is worth mentioning that histogram and wavelets measurements depend on MRI signal types: e.g. “spin–lattice” relaxation time (T1 or T1 weighted image); “spin–spin” relaxation time (T2 or T2 weighted image); proton density (PD) (see Additional file 2: Dataset 2, Additional file 3: Dataset 3 for additional details).

### Index of focus

Focus index or index of focus (abbreviated: I-focus) is neither an integral parameter in MaZda nor in B11. It has been scarcely used before to measure perceived quality and realness of wood images [56], an embellishing technique for enhancing appearance of floor murals, backdrops and wall decorations used in low-cost construction. Focus index is calculated by dividing skewness by kurtosis. Skewness is a parameter which measures surface symmetry or lack of symmetry, imperfections, scanner misalignment. It indicates the even portion of a surface and the direction of distortion in the uneven portions (negatively and positively skewed). On the other hand, kurtosis is a measurement of the peakedness and flatness of fine details and edges where textural variations occur. When an image is in focus, edge elements or objects are sharp. Focus index combines these two parameters to thus assess quality by measuring focal or perspective distortion and sharpness. An image may be sharp but distorted and vice versa. In magnetic resonance imaging, focus index varies from one perspective plane to another (i.e. axial, coronal, sagittal). Ideal focus index is zero or close to zero [56, 57]. Sharpness quality for focus index values outside the range of [−1, +1] can be easily perceived by the naked, normal human eye. It is also important to note that focus index alone does not determine image quality. The common pitfall of the skewness and kurtosis is their sensitivity to artifacts like noise, grain, post-editing sharpening and Gaussian blur [56, 57].

### Index of dispersion

Index of dispersion [also known as variance-to-mean ratio (VMR)] is a measurement used to determine the clustering or dispersion of a luminance. In this research, VMR was utilized to quantify the volatility (difference) of each individual ROI (ventricle, thalamus, grey matter, white matter). The larger the difference between the coefficients of dispersion (dispersion index values) the greater the variability between the ROIs. Variance is the VMR component which measures contrast and boundaries (edges) in a surface. Like kurtosis and skewness, variance is also affected by noise [58]. Variance is the difference in luminance and/or color that makes an object or its representation in an image or a ROI to display distinguishable. In other words, variance is a histogram parameter which can be used to determine contrast volatility in an image. Finally, mean is a reflection of the average grayscale luminance in MR images. It is directly proportional to the stored bits of an image.

### Resolution

3 T scanner is by default constructed to capture more details than 1.5 T. Its higher magnetic strength, when used properly, can also overcome problems with signal-to-noise ratio (SNR). Therefore, 3 T units can conditionally produce image with more information and less distortion. This superiority is technically due to denser sampling of K-space for the same size of field of view (FOV), resulting in increased resolution. Nonetheless, several other factors affect the visual interpretation of the MR image which reaches the radiologist via medical-diagnostic LCD (e.g. acutance, physical and technical unit settings, SNR, FOV, extended knowledge of elements affecting perceived appearance, realness, sharpness, etc) [59]. “Sharper” does not always signify “more details” and “more details” does not always signify “sharper.” Quality depends on more factors than just “more details”. A MR image can have “higher resolution” and “more details” and be not sharp or be degraded by artifacts and thus of poor quality. Unless its quality can be restored with computer vision and/or editing tools, such an image would be useless for live medical application. In clinical practice, MR units have been used to detect anatomical, functional and molecular anomalies. MR imaging modalities and the quality of their products are crucial because physicians not only rely on them to reach a firmly conclusive diagnosis but also to make final interpretation to administer therapeutic care; which if wrong can lead to malpractice litigation.

### Fisher coefficient

One of the most promising application of texture analysis in medicine is early detection of tumorous signs and prophylaxis of cancer. A methodology of interest to this research was Fisher texture analysis. It has been used before in medical research to discriminate healthy from benign or malignant tissue. In this experiment, it was necessary to develop a modified calculation of Fisher coefficient—coined as “feature-selection coefficient” (Eq. 1). It was derived from an amalgamation of Fisher coefficient and statistical principles of analysis of variance (ANOVA).

1 $$F = \frac{{D^{2} }}{{V^{2} }} = \frac{{\frac{1}{{1 - \sum\nolimits_{k = 1}^{K} {P_{k}^{2} } }}\sum\nolimits_{k = 1}^{K} {\sum\nolimits_{j = 1}^{K} {P_{k} P_{j} \left| {\mu_{k} - \mu_{j} } \right|^{2} } } }}{{\sum\nolimits_{k = 1}^{K} {P_{k} V_{k}^{2} } }}$$

In Eq. 1, feature-selection coefficient is derived from Fisher coefficient and ANOVA, where D is between-class variance, V within-class variance, P_k_ probability of feature k, V_k_ variance value of feature k in given class and μ_k_ mean value of feature k in given class (see MaZda user manual, http://www.eletel.p.lodz.pl/mazda/download/mazda_manual.pdf for further details).

Resolution already indicated that 3 T captured more details than 1.5 T. However, it was still crucial to test the differential capability of each modality in their own defined three-dimensional (3D) space (with dispersion index, focus index, wavenhl) and the effects of image compression on individual parameters. Features extracted from MaZda histogram were plotted on B11 XYZ space along with two controls [3T: C_min_ (0, −1, 0), C_max_ (5500, 1, 120,000); 1.5 T: C_min_ (0, −1, 0), C_max_ (307, 1, 120,000)]. Note that only the x-axis is different. This comparative method ensured that 1.5 and 3 T are fairly measured within the resolution limit of the tested samples. The B11 program computed F from ANOVA derived parameters (F = D2/V2, where D = between-class variance and V = within-class variance). Then it placed ventricles, thalamus, grey matter, and white matter within a 3D space. By themselves, C_min_ and C_max_ yielded a maximum F of 10E + 6 and a misclassified data error (MDE) of 0% for both 1.5 and 3 T. The effect of sample-mismatching on F and the software mechanics were closely studied with control parameters and 3D graphs. It was observed that changing one C_max_ to [0, −1, 60,000] while others remain constant caused a 25% MDE and F value dropped to 18.

## Results

**Fig. 9:**
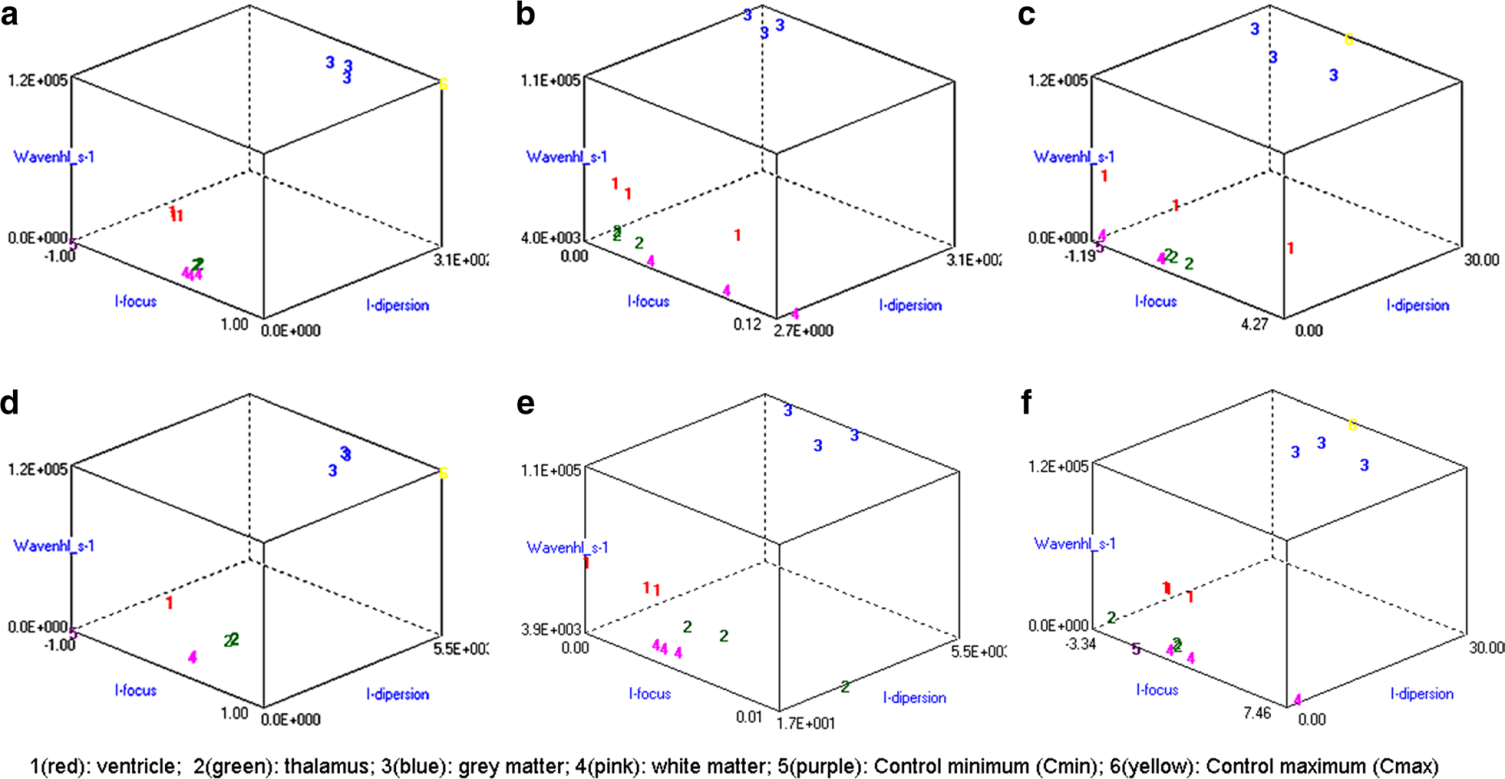
Graph showing difference between ROIs. Raw-data analysis was performed to compute F with 1-nearest-neighbor (NN) classification and no feature standardization. Same samples and ROIs were used in both pre- and post- image compression. **a** 1.5 T uncompressed DICOM; Fisher coefficient computation with controls: Cmin = (0, −1, 0); Cmax = (307, 1, 120,000); F = 426.0; MDE = 0%. **b** Same 1.5 T samples: zoomed in, mostly on the *y-axis*. **c** same 1.5 T after compression to 8 bits; Fisher coefficient computation with controls: Cmin = (0, −1, 0) Cmax = (30, 1, 120,000); F = 776.0; MDE = 5.56%. **d** 3 T uncompressed DICOM: F = 1787.0; MDE = 0%. **e** Same 1.5 T samples: zoomed in, mostly on the *y-axis*. **f** Same 3 T after compression to 8 bits. 3 T: F = 2344.3; MDE = 0%

Wavenhl, focus index (skewness-to-kurtosis ratio) and dispersion index (variance-to-mean ratio) reveal better quality for 3 T (Fig. 9). Though both 1.5-T and 3-T images were 16-bit DICOM encoded, nearly 16 and 12 usable bits were measured in 3-T and 1.5-T images, respectively. Four bits in all 1.5 T images were padded. Such K-space encoding methods appeared to reduce noise, by adding illusionistic details which are not really part of the image. In contrast, all 3 T images were zero-bit padded. This encoding technique provides space for storing more details and increases the likelihood of noise but as well as edges—which in turn are very crucial for differentiation of closely related anatomical structures. Both encoding modes are possible with both units, but higher 3 T resolution is the main difference. Apart from surprisingly larger Fisher coefficient (Fig. 9), no significant parametric difference was observed (p > 0.05) when DICOM files with 12 and 16 stored bits were degraded to 8-bit BMP (Additional file 2: Dataset 2). MaZda measured an equal detrimental loss of quality in both 1.5-T and 3-T BMP images (Additional file 1: Dataset 1); but ROI discrimination was still better in 3 T. In both pre- and post- compression, F was larger for 3 T. Unexpectedly, F was even better in the degraded images.

### Supplemental trial runs

**Fig. 10:**
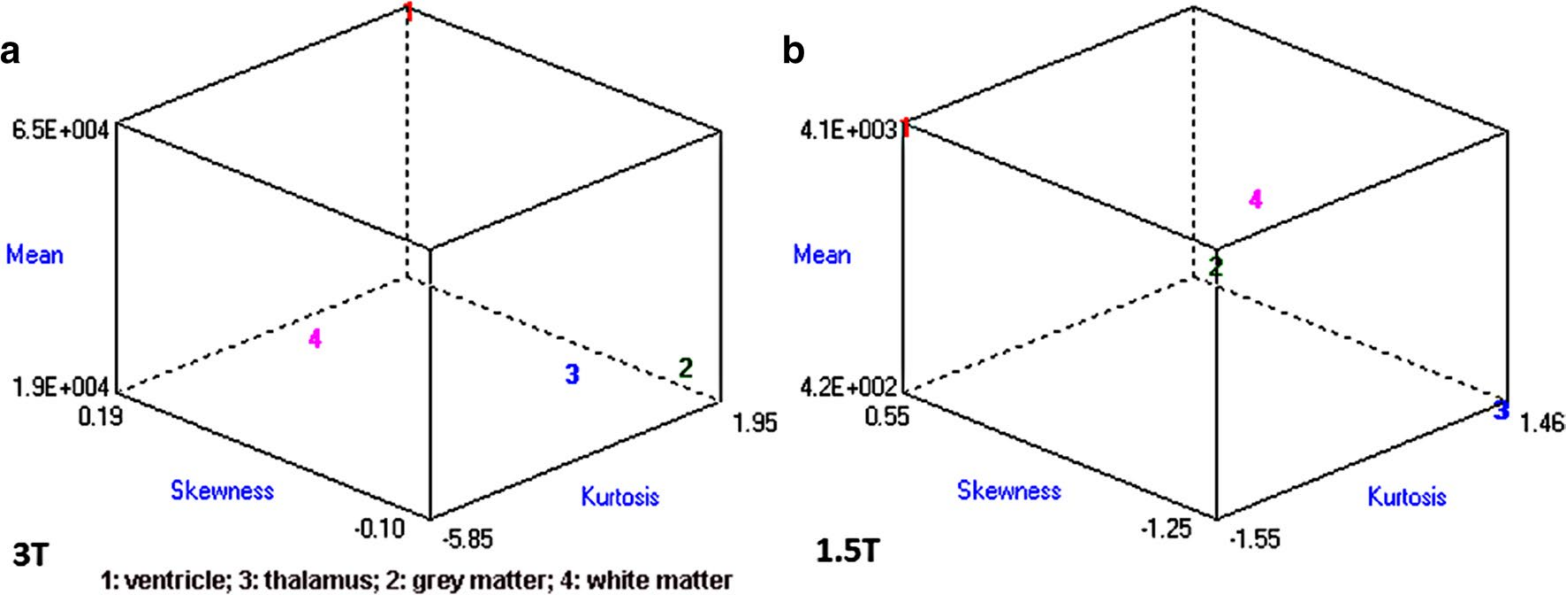
**a** Difference between ROIs for 3 T with two images from same T2 sequence (same patient). *1* ventricle; *3* thalamus; *2* grey matter; *4* white matter. **b** Difference between ROIs for 1.5 T with two images from same T2 sequence (same patient). *1* ventricle; *3* thalamus; *2* grey matter; *4* white matter

A preliminary trial was carried out as per recommendations in MaZda and B11 user manuals/tutorial guides (Fisher coefficient computation). The manufacturer’s recommended method was tested with two different frames (images), extracted from a single 3 T sequence and two different frames from a single 1.5 T sequence. Then two overlapping ROIs were selected and grouped—per anatomical structures in each image (i.e. 2 ROIs of thalamus, 2 ROIs of ventricles, and so on). The outcome is delineated later in this section herein. It is also important to note that some aspects of this research were out of the researchers’ control—meaning that radio-technicians performed MRI examinations that attending physicians and hospitals deemed necessary and safe for their patients. Thus the images collected were limited to the setting modes delineated in the MRI prescriptions and hospital-approved imaging protocols—which in turn is contingent upon routine checkups, suspected pathologies and gestational age—as well as regional-clinical practice. The latter varies from one location to another. In the USA, for example, Food and Drug Administration (FDA) does not approve MRI with intravenous gadolinium-based contrast agents for use during pregnancy—although nobody has yet discovered any hazards to fetus. A slice thickness of 3 mm is commonly used for routine fetal brain examinations, while 4-mm is used for other organs. In all collected samples, magnetic resonance imaging was performed as an adjunct to ultrasound for clarification of conditions mostly not related to brain anomalies. Throughout this research, the selection consisted of 1.5 and 3 T sequences which were within the acceptable specifications: no apparent brain defects; slice thickness: 3–5 mm; repetition time: 800–2100; echo time: 1–95; imaging frequency: 60–130; pixel bandwidth: 400–1200. Final sorting step necessitates pixel-by-pixel processing of 16-bit grayscale and then identification of parameters with lowest standard deviation. What does this sorting algorithm entails. It means that all MaZda parameters limited to 8-bit and 12-bit feature extraction were excluded. From the parameter list matching the criteria, three (skewness, kurtosis, and mean) were chosen for the B11 tests because the measurements were fixed every time the process was repeated and had lowest standard deviations between frames. Furthermore skewness, kurtosis, and mean parameters had measurements that were very close to the average values. These extracted features were then analyzed in 3D space with B11. For images extracted from the same 1.5 and 3 T sequences, RAW analysis yielded a maximum Fisher coefficient of E + 5 and perfect 0% misclassification error for both imaging modalities. B11 plugin was used to generate and examine the result on a graph (Fig. 10). Though the graph appears as texture analysis of a single image, each number is actually from two different frames overlapping in three-dimensional space. The software guidelines as well as its engineers were consulted regarding B11 limitation. It turned out that B11’s Fisher Coefficient (F) is internally computed from modified ANOVA statistics—by customizedly dividing a nominator (difference between regions) over a denominator (difference within regions). We did not investigate further and neither modify the computation mechanics of Fisher Coefficient, as the software license did not authorize us to do so. Larger F was construed to mean that there is more difference between ROIs. For the purpose of this research, straight-forward comparative test with Fisher coefficient method was inconclusive, as F was the same for comparison between 3 and 1.5 T ROIs (see Additional file 3: Dataset 3—appendix 1, appendix 2, appendix 3 for further details).

**Fig. 11:**
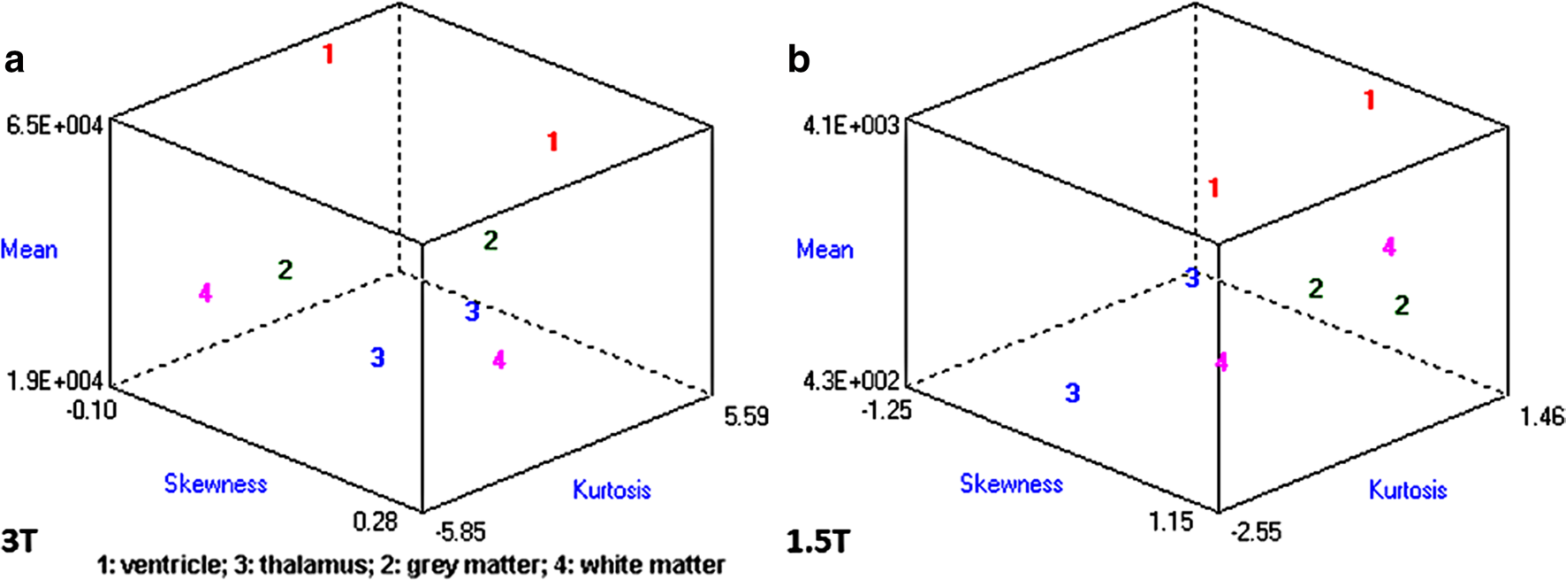
**a** Difference between ROIs for 3 T with two images from different T2 TSE [Turbo spin echo sequences (different patients)]. *1* ventricle; *3* thalamus; *2* grey matter; *4* white matter. **b** Difference between ROIs for 1.5 T with one image from PD and one from T2 HASTE sequence (different patient). *1* ventricle; *3* thalamus; *2* grey matter; *4* white matter

The software maker recommended to use different frames from different MR sequences and/or studies. Different sequences from different patients were scored (Fig. 11). Fisher coefficient was 897.4 for 3 T and 144.1 for 1.5 T. It is important to note that proton density (PD) sequence is very different from T2. The trial was re-run with two 1.5 T T2 HASTE (half-Fourier acquisition single-shot turbo spin-echo) sequences from two different patients; and F was 448.6. The software maker did not have an immediate solution for the B11 limitation and thus recommended to use the software when comparatively testing many images from different sequences.

In technically controlled settings, the software maker suggestions are feasible. However, in medical practice, it is not a viable process. It would be detrimental to get so many MR studies from a single patient for ROI analysis.

### Grayscale quantification with histogram-based parameters

There are misconceptions about the usefulness of grayscale quantification and its applications in medicine. While this area of diagnostic radiology is still emerging and under-documented—grayscale texture analysis with histogram-based parameters is already proven to be very useful for differentiation of benign from malignant thyroid cancer, for example. Such parameters can be used to detect micro-calcifications inside nodules—which is an early sign of thyroid malignancies. In this research, histogram parameters was used to calculate focus index ratios (skewness-to-mean kurtosis ratio) and dispersion index values (variance-to-mean ration). What is the logical meaning of using such parameters in magnetic resonance imaging? Histogram-based parameters don’t just generically measure intensity of grayscale and signal-to-noise ratio (SNR). Such parameters can also be used for morphometric measurements, texture segmentation and discrimination. The functionality of focus index can be simply understood from its numerator and denominator. In computational- visual cognition and imaging statistics, skewness is a numerical measurement of symmetry and dissymmetry in an image. Kurtosis, on the other hand, is a histogram parameter that is sensitive to Gaussian blur and out-of-focus blurs (indirect sharpness), noise and grain (simulated sharpness), peakedness and flatness of fine details and edges (due to image resolution), chromatic aberration blur (optical distortion) and other artifacts. Focus index would probably sounds novel to most or perhaps all readers of this article. Yet, it is sparsely used in reality-rendering imaginary to measure realness (naturalness) of surface perception—and thus the phenomenon that human eye does not always sees what it thinks it does. Applications of focus index include photo-realistic rending, optical/visual illusion, methatetic continuum (change in stimulus quality) and indiscernible visual perception between natural materials and replicas (e.g. outdoor backdrops, floors and murals) used in filmmaking and architectural construction. A pitfall with focus index is its susceptibility to digital and mechanical artifacts recorded by imaging equipment but not really part of the image. How was the problem solved? Perceived quality of photo-realistic prints was previously documented to be optimally construed as real—when focus index is zero or nearly zero but within −1 and +1 interval. Furthermore focus index and dispersion index were concomitantly used to rule out noise (artifacts) from true signal (actual anatomy). The definition of dispersion index can be technically interpreted in the difference between its dividend and divisor as well as the resulting quotient. Variance is a numerical measurement of contrast and captured details in an image; and thus it is affected by resolution. Mean is the average luminance derived from stored bit value in an image (ROI in this case). Mean may be misunderstood as a mere measurement of pixel values, but herein it also served as a reference value for the variance. Stored bit is not to be confused with allocated bit and high bit. The stored bit value reported in the DICOM tag did not always correspond to that measured with MaZda. A large gap between the variance and the mean could be an indication of fine details or edges (e.g. anatomical boundaries or pattern changes within anatomical structures); or it could also be due to randomly occurring noise and/or unwanted artifacts. Both 1.5 and 3 T images were encoded in 16-bit DICOM container. Why don’t they have the same quality? Not all bit slots necessarily contain true details (actual anatomy). In our test, converting (uncompressing) 16-bit Lossless JPEG DICOM to 16-bit RAW DICOM did not improve quantification of bit slots which actually contain anatomical details—though the RAW file was larger. If stored bit was initially 12, it was still 12 after uncompression. MR scanner settings are also known to digitally and mechanically affect quality. Therefore, 3 and 1.5 T images were closely matched by matrix size, field-of-view (FOV), slice thickness, phase and frequency encodings. Besides magnetic strength and speed, the quality of captured details also depends on the voxel size and thus the resolution of 1.5 and 3 T (in the collected samples, 256 × 256 and 446 × 446 respectively). Separating noise and discretization artifacts from true signals is a time-consuming editing process. A more efficient solution was to get rid out of heavily noisy samples. So doing does not necessarily imply that we think that noise is always bad. Despite its degradative nature, noise can also improve visual appearance of an image. Dither, for instance, is in-machine (real-time) or post-editing added noise to filter some unwanted artifacts (e.g. off-resonance bandings, posterization)—in order to improve acutance (sharpness) and therefore visual perception. By narrowing the reference range of the focus index quotient to [−1, +1], a large number of noisy samples were excluded, as a result of displaying characteristics of random quantum mottle (grainy appearance). Hence the larger variance observed in 3 T parametric measurements are likely due to sharply captured anatomical details with fine edges—rather than digitally and mechanically generated noisy artifacts.

### Computation of difference between ROIs

**Fig. 12:**
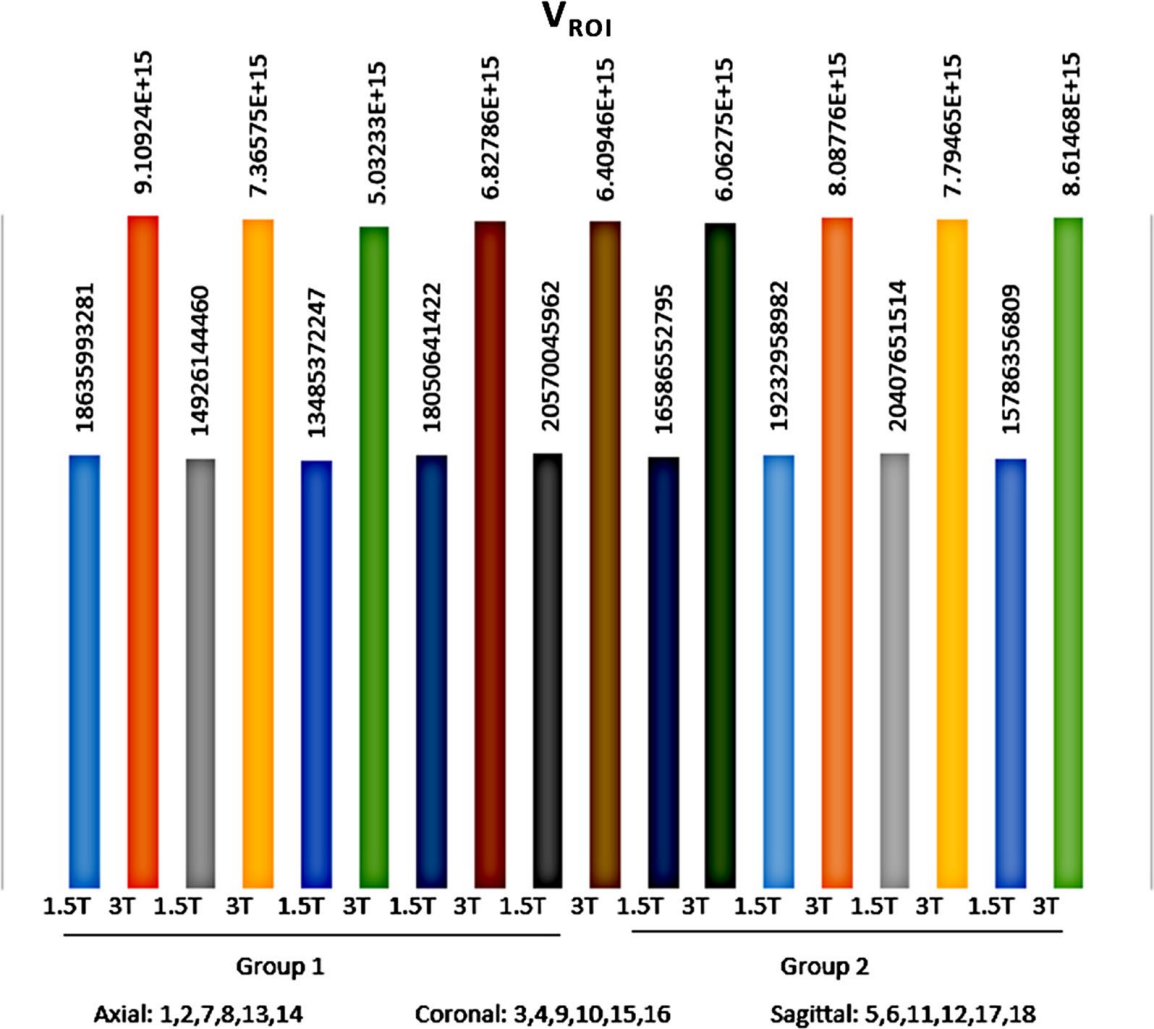
ROI variability graph showing difference between 1.5 and 3 T

**Fig. 13:**
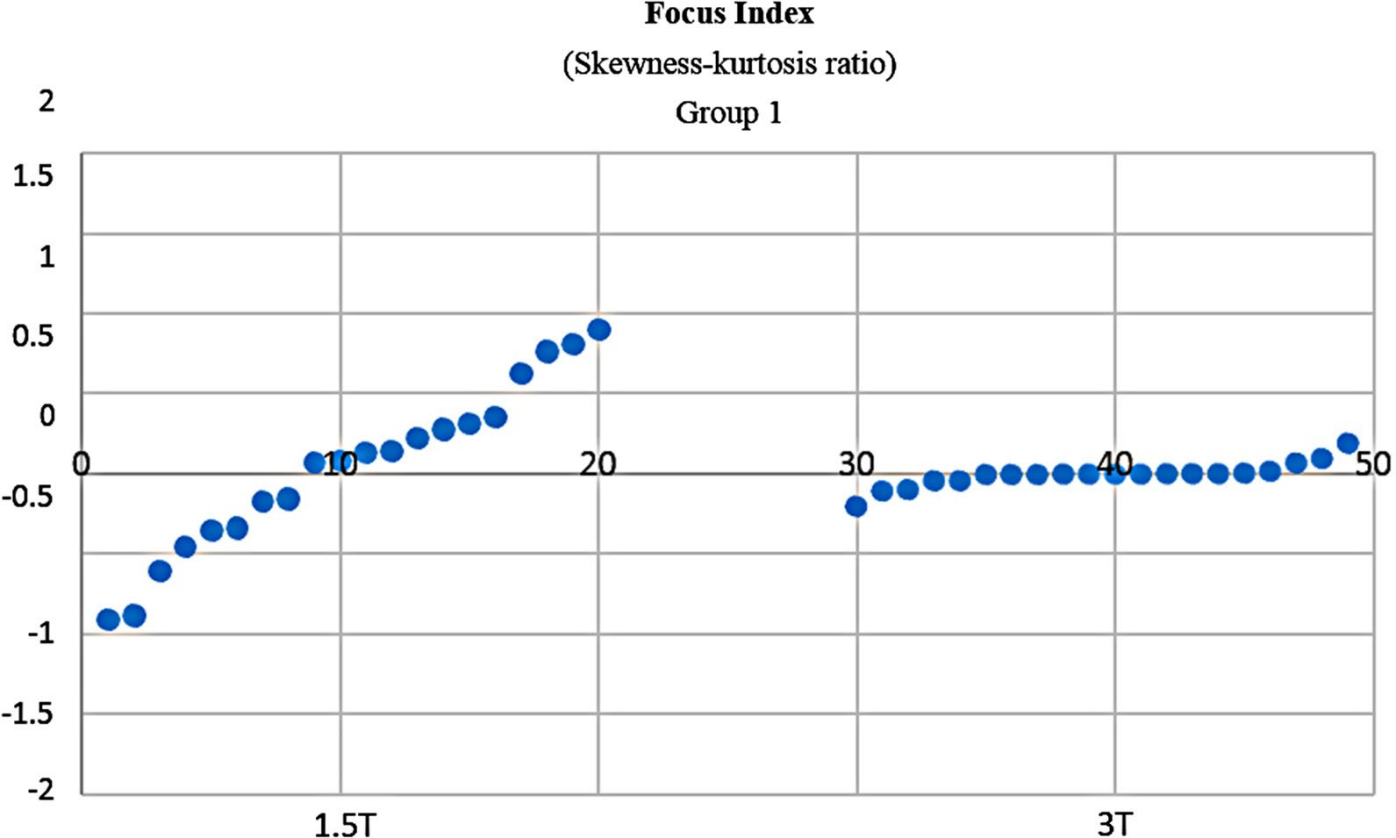
Sharpness distribution: *Group 1* acceptable range is [+1, −1]. Zero is absolute sharpness; in other words, better focus. 3 T has more points closer to zero

**Fig. 14:**
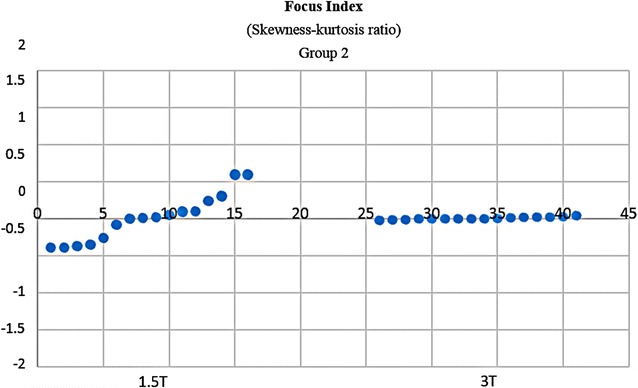
Sharpness distribution: *Group 2* acceptable range is [+1, −1]. Zero is absolute sharpness; in other words, better focus. 3 T has more points closer to zero

**Fig. 15:**
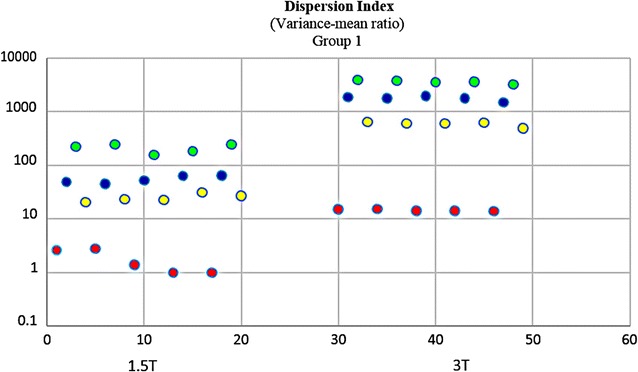
Statistical dispersion: *logarithmic* graph (Group 1) showing difference between 1.5 and 3 T. 3 T ROIs are more spread out; thus better for distinguishing close anatomical structures in fetal MRI. Regions of interest—*red:* ventricle—*yellow:* white matter—*blue:* thalamus—*green:* grey matter

**Fig. 16:**
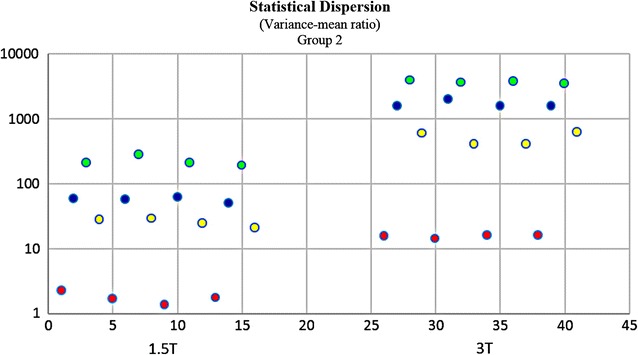
Statistical dispersion: *logarithmic* graph (Group 1) showing difference between 1.5 and 3 T. 3 T ROIs are more spread out; thus better for distinguishing close anatomical structures in fetal MRI. Regions of interest—*red:* ventricle—*yellow:* white matter—*blue:* thalamus—*green:* grey matter

It would be subjective to just judge the quality by simply comparing 1.5 and 3 T images on histogram graphs. As a remedy, other comparative tests were developed and carried out. In Fischer coefficient, the numerator (difference between the ROIs) was the quantification wanted. Hence analysis of variance was subsequently performed to quantify the difference between 3 and 1.5 T ROIs. The parameters were then imported into STATISTICA version 10 for further statistical processing and analysis. All the results were exported to excel, and a dataset was generated (Additional file 4: Dataset 4). 3 T images (~E + 15) had much higher ROI variations than 1.5 T images (~E + 10). In terms of parametric quantification, the increased variability between ROIs revealed that 3 T is a better discriminative tools than 1.5 T (Fig. 12). Focus index values and dispersion index values were used respectively to measure sharpness distribution and statistical dispersion (Figs. 13, 14, 15, 16). The focus index graph shows that 3 T images have better sharpness and better focus in both groups. As per dispersion index graph, 3 T is also better in terms of spatial resolution. In all measurements, the results were statistically significant with p < 0.01 (see Table 2).

**Table 2:** The best nine values were selected to determine p values

I_FOCUS_				I_DISPERSION_			
1.5 T G1	3 T G1	1.5 T G2	3 T G2	1.5 T G1	3 T G1	1.5 T G2	3 T G2
0.174816	0.001664	0.261094	0.013746	245.8439	3845.506	272.6785	3919.487
0.155916	0.001614	0.076755	0.001496	241.6155	3725.265	208.9656	3684.96
0.07019	0.000614	0.002159	0.000435	222.8727	3584.081	207.59	3605.269
0.084207	0.000317	0.010039	5.16E−07	183.2519	3518.256	191.5476	3396.184
0.12892	0.000824	0.018455	3.76E−06	152.8732	3234.123	62.18858	1969.419
0.14576	0.001041	0.053909	0.00046	63.68822	1949.152	59.11231	1557.758
0.222427	0.001126	0.095316	0.00049	62.51141	1861.574	56.22063	1547.73
0.275593	0.002816	0.100121	0.004151	52.44858	1775.027	50.28433	1542.073
0.315591	0.003521	0.239347	0.01374	48.76635	1763.935	28.30434	614.1261
m = 0.175	m = 0.0015	m = 0.0952	m = 0.0038	m = 142	m = 2806	m = 126	m = 2426
sd = 8E−02	sd = 1E−03	sd = 9E−02	sd = 5E−03	sd = 85	sd = 935	sd = 92	sd = 614
t(16) = 6.3 p = 0.0001		t(16) = 2.9 p = 0.011		t(16) = −8.5 p = 0.0001		t(16) = −5.6 p = 0.0001	

## Discussion

In this experiment, the findings revealed that 8-bit BMP actually yielded higher F than the original 16-bit DICOM. Larger F coefficient does not necessarily mean better quality. Nonlinear discriminant analysis (NDA), linear discriminant analysis (LDA), and principal component analysis (PCA) can generate low or large F when B11 is overtrained [60]. Thus RAW data analysis was performed, as it is more constant than NDA, LDA, and PCA transformations. With featured standardization turned-off, RAW data analysis calculated same F values each time measurements are repeated. In this case, larger F from 8-bit BMP is unlikely to be due to overtraining problem in the network. It is rather due to squeezing tricks employed in lossy compression. While ROIs appear more uniform, some fine details get discarded. In MaZda texture analysis software, histogram can indeed measure regional tonality directly from image statistics rather than from secondary visual means such as graphical appearance on a monitor screen; which is susceptible to eye limitations (e.g. acutance, screen size, etc). Thus 16-bit image can be accurately mapped three-dimensionally from intensity value of 0 to a maximum intensity of 65,535; and variance provides information about details, sharpness and edges in the image. 3 T is technically able to capture more details due to higher resolution. Nevertheless, higher resolution does not always guarantee better quality and neither does it always mean more details. Without proper unit settings to booster available dynamic range, resolution is just a mere increment in the cost of storage media. Available dynamic range of an image is the information that is not seen until it is actually used. The original encoding bit space is crucial for storage of captured details. In 8-bit space, for instance, it is mathematically impossible to recover fine highlight details such as blue sky in an indoor image of a blown-out window on a bright sunny day. Lowering exposure shows grey sky. In 12- or higher-bit space, the sky should be still blue. This is where available dynamic range comes in play. Wavelets, on the other hand, were not used for measuring quality but for double-checking and matching regions of interest (i.e. ventricles, thalamus, grey matter, and white matter). Concomitant texture analysis with wavelets and histogram allows accurate classification of closely related anatomical structures in the brain during development, and thus an important set of tools for early detection of tissue changes in the brain. For example, grey matter is vastly present in the brain. It can be difficult for the unaided human eye to trace the boundaries of hypothalamic nuclei on a prenatal magnetic resonance image. It is due to the fact that grey matter is also present deep inside the cerebrum and inside the thalamus, hypothalamus, and the basal ganglia. Even with the fetal brain fully formed, it is still elusive for the human eye to delineate and differentiate these anatomical structures during pregnancy. In the overexposure example, the human eye would see the exact same blown-out region with no obvious quality discrimination in either 8-bit or 16-bit space, while histogram and wavelets would pick up the hidden details and reveal a difference in parametric values. This is where texture analysis software fits in biomedical imaging.

### Constraints, limitations, and assumptions

MaZda Texture Analysis version 5 was used throughout this experiment (Additional file 5). It is a quantitative analysis tool which can extract various parameters from 16-bit DICOM without need of conversion to 8-bit BMP, and it can measure the actual stored bits within a DICOM container of 16 allocated bits. This feature is crucial in terms of comparing 1.5–3 T for discriminative power of closely related anatomical structures in fetal brain imaging. Unfortunately, many MaZda parameters were excluded from this experiment because of either 8-bit or 12-bit limitations. An updated, customized version of this software package would require several man-hours and therefore was not feasible in due time.

### Recommendations

As of today, there are still numerous talks about the usefulness of 3 T MRI in medical practice and the possibility of deleterious effects during early pregnancy. The scientific views about the differential quality between 1.5 and 3 T are mixed and perplexed due to subjectivity and unreliable interpretative techniques, which largely depend on the human eye perception. Observational studies have reported that the greater magnetic field of 3 T produces sharper images based on visual appearance. Such findings are subjected to controversion because different pairs of eyes don’t always see the same thing. The normal human eye was reported to have a differential power of a quasi 8-bit scanner. Very few people perceive details beyond that limitation. From a financial point of view, billions of dollars could be saved if 1.5 T is used instead of 3 T MRI. Nevertheless, financial standpoint and hardship may indeed prevent a clinician from ordering 3 T MRI, instead of using relevance to beneficiaries as a guide to make such a decision. Some still raise questions over the safety of 3 T magnetic field in medical practice. Nevertheless, twelve years ago, studies have already shown that 8T MRI causes no obvious damage to the human body. Like other imaging modalities, the main issue in the medical community that is affecting the interpretation of 3 T MR images is subjectivity and observer dependence. It is a challenging task for different human eyes to correctly identify square-pixel variations between the gray-scale image presented in Fig. 1. A key feature of texture analysis with computer vision is that it can quantify texture in pixelated 1.5 and 3 T MR images into numerical values, which in turn can be used to assess image quality—therefore eliminating subjectivity and reproducibility issues in diagnostic imaging. Some of our guest reviewers had argued that texture quantification was not necessarily an advantage. Yet the World Health Organization (WHO) has noticed a fall in misdiagnosis and treatment cost and a rise in detection and diagnosis of the pandemic disease of tuberculosis (TB)—with introduction of computer-aided interpretation software in regions with prevalent TB outbreak. Again, this research is not about whether human vision is better than artificial vision or vice versa. We disclaim any so-construed allegations. The goal is that texture analysis may assist obstetricians and radiologists in making more accurate and objective medical diagnosis of prenatal pathologies. Reaching this outcome is a matter of testing and finding suitable artificial systems and methods of texture analysis for the appropriate set of images. Last but not least, artificial magnetic fields of 1.5 T (30,000 times greater than earth’s magnetic field) and 3 T (60,000 times greater than earth’s magnetic field) [61], scanners have raised theoretical concerns in the medical community. Unknown risks such as teratogenic and biological effects—if they really exist—could be reduced by using 1.5 T during the first 28 weeks—as its quality is sufficient [62–67].

## Conclusions

Unquestionably, 3-T exhibits better quality than 1.5-T fetal magnetic resonance imaging. The results were significant (p < 0.05). Nevertheless, 1.5-T is of sufficient quality for routine fetal examination during early pregnancy and therefore can lower the risks of unknown teratogenic effects. Though 3 T fetal MRI exhibits superior image quality, its usefulness, health claims, side effects, benefits, and safety demand further investigation.

## Additional files

**Additional file 1: Dataset 1.** Figure 8 data: stored bits in 1.5/3 T DICOM/BMP, measured with MaZda version 5.

**Additional file 2: Dataset 2.** Effects of pull-down conversion: 12/16-bits grayscale DICOM to 8-bit BMP (RGB-24).

**Additional file 3: Dataset 3.** Raw texture analysis/Fisher coefficient: ➤ appendix 1, ➤ appendix 2, ➤ appendix 3.

**Additional file 4: Dataset 4.** Data for supplemental trial runs: 36 × 1.5 T; 36 × 3 T; group 1: 20–28 weeks; group 2: 29–40 weeks.

**Additional file 5.** MaZda Package v5 RC HG (release candidate: Hugues Gentillon): this pre-release (beta) version contains: 1) MaZda version 5 for achromatic and chromatic image analysis, 3D editing, feature extraction & selection, geometrical features for ROIs; 2) B11 version 3.3 for feature clustering, image segmentation, unsupervised & supervised classification.

## Abbreviations

2D: two-dimensional
BMP: bitmap
CNS: central nervous system
ROI: region of interest
CAD: computer-aided diagnosis
COST: cooperation in science and technology
DICOM: digital imaging and communications in medicine
E+: positive exponent
E-: negative exponent
JPEP: Joint Photographic Experts Group
LCD: liquid crystal display
HG: Hugues Gentillon
LS: Ludomir Stefańczyk
MS: Michał Strzelecki
MRL: Maria Respondek-Liberska
MR: magnetic resonance
MRI: magnetic resonance imaging
PD: proton density
RAW: reas as written
RGB: red, blue, green
RGB-24: color space with 8 bits per channel (8 × 8 × 8)
ROI: region of interest
US: ultrasound
USG: ultrasonography
T: tesla
TB: tuberculosis
V_ROI_: variations between ROIs
Wk: week
