# Supplementary material for: Parameter set for computer-assisted texture analysis of fetal brain
Source: BMC Res Notes. 2016 Nov 25;9:496. doi: 10.1186/s13104-016-2300-3 (PMC5124296; doi:10.1186/s13104-016-2300-3)
Supplement: Supplementary file 1 — Additional file 1: Dataset 1. Figure 8 data: stored bits in 1.5/3 T DICOM/BMP, measured with MaZda version 5. [file 13104_2016_2300_MOESM1_ESM.zip › dataset 1_Parameter set for Computer-Assisted Texture Analysis of Fetal Brain.pdf]

Dataset 1: fig 6 data: stored bits in 1.5/3T DICOM/BMP, measured with MaZda version 5

3T DICOM	1.5T DICOM	1.5T BMP	3T BMP
**65530**	4090	255	255
**65102**	4095	255	255
**54153**	4091	255	255
**58645**	4092	255	255
**48596**	4095	255	255
**52365**	4095	255	255
**54514**	4090	255	255
**62658**	4087	255	255
**65125**	4090	255	255
**65458**	4090	255	255
**65535**	4091	255	255
**65502**	4052	255	255
**65535**	4057	255	255
**61256**	4044	255	255
**65235**	3948	255	255
**62593**	4015	255	255
**64852**	4023	255	255
**65028**	4058	255	255
**65120**	4078	255	255
**65289**	4015	255	255
**64215**	4069	255	255
**65245**	4058	255	255
**60152**	4025	255	255
**58962**	4015	255	255
**58562**	4056	255	255
**58964**	4058	255	255
**61253**	4085	255	255
**63258**	4026	255	255
**64215**	4023	255	255
**65352**	4058	255	255
**65259**	4027	255	255
**62658**	4059	255	255
**65125**	4056	255	255
**65458**	4079	255	255
**65535**	4012	255	255
**65502**	4036	255	255
**65535**	4019	255	255
**61256**	4018	255	255
**65235**	4037	255	255
**62593**	4016	255	255

**64852**	4017	255	255
**65028**	4019	255	255
**65120**	4018	255	255
**65289**	4015	255	255
**64215**	4016	255	255
**65245**	4013	255	255
**65028**	4089	255	255
**65120**	4095	255	255
**65289**	4092	255	255
**64215**	4091	255	255
**65245**	4095	255	255
**60152**	4095	255	255
**58962**	4095	255	255
**58562**	4095	255	255
**58964**	4090	255	255
**61253**	4023	255	255
**63258**	4058	255	255
**64215**	4027	255	255
**65352**	4059	255	255
**65259**	4056	255	255
**62658**	4079	255	255
**65125**	4012	255	255
**65458**	4036	255	255
**65535**	4019	255	255
**65502**	4018	255	255
**65535**	4015	255	255
**61256**	4016	255	255
**65235**	4013	255	255
**58596**	4089	255	255
**52365**	4095	255	255
**55259**	4092	255	255
**52658**	4091	255	255
**65125**	4085	255	255
**65458**	4026	255	255
**65535**	4023	255	255
**65502**	4058	255	255
**65535**	4027	255	255
**61256**	4059	255	255
**65235**	4056	255	255
**62593**	4079	255	255
**64852**	4012	255	255

**65028**	4036	255	255
**65120**	4091	255	255
**65289**	4052	255	255
**64215**	4057	255	255
**65245**	4044	255	255
**60152**	3952	255	255
**58962**	3869	255	255
**58562**	3895	255	255
**58964**	3795	255	255
**61253**	3689	255	255
**63258**	3896	255	255
**64215**	4019	255	255
**65352**	4012	255	255
**55259**	4036	255	255
**52658**	4058	255	255
**65125**	4015	255	255
**65458**	4091	255	255
**58962**	4027	255	255
**58562**	4059	255	255
**58964**	4056	255	255
**61253**	4079	255	255
**63258**	4012	255	255
**64215**	4036	255	255
**65352**	4091	255	255
**55259**	4052	255	255
**65152**	4057	255	255
**65125**	4044	255	255
**65458**	3952	255	255
**65535**	3869	255	255
**65502**	3895	255	255
**65535**	4058	255	255
**61256**	4027	255	255
**65235**	4059	255	255
**58596**	4056	255	255
**52365**	4079	255	255
**55259**	4012	255	255
**52658**	4036	255	255
**65125**	4019	255	255
**65458**	4018	255	255
**65535**	4037	255	255
**65502**	4016	255	255

**65535**	4017	255	255
**61256**	4019	255	255
**65235**	4018	255	255
**62593**	4015	255	255
**64852**	4016	255	255
**65028**	4013	255	255
**65120**	4089	255	255
**65289**	4095	255	255
**64215**	4092	255	255
**65245**	4090	255	255
**60152**	4087	255	255
**58962**	4090	255	255
**65502**	4090	255	255
**65535**	4091	255	255
**61256**	4052	255	255
**65235**	4057	255	255
**62593**	4044	255	255
**64852**	4018	255	255
**65028**	4089	255	255
**65253**	4095	255	255
**62559**	4058	255	255
**64215**	4095	255	255
