# Supplementary material for: Parameter set for computer-assisted texture analysis of fetal brain
Source: BMC Res Notes. 2016 Nov 25;9:496. doi: 10.1186/s13104-016-2300-3 (PMC5124296; doi:10.1186/s13104-016-2300-3)
Supplement: Supplementary file 3 — Additional file 3: Dataset 3. Raw texture analysis/Fisher coefficient: ➤ appendix 1, ➤ appendix 2, ➤ appendix 3. [file 13104_2016_2300_MOESM3_ESM.zip › dataset 3_appendix1_Parameter set for Computer-Assisted Texture Analysis of Fetal Brain.pdf]

APPENDIX 1

Dataset 3: Raw texture Analysis / Fisher Coefficient

Column1	Column2	Column3	Column4	Column5	Column6	Column1	Column2	Column3	Column4	Column5
MaZda report for fig. 3		**Difference between ROIs for 3T.**				MaZda report for fig. 4		**Difference between ROIs for 1.5T.**		
*features						*features				
1 Kurtosis						1 Kurtosis				
2 Skewness						2 Skewness				
3 Mean						3 Mean				
*categories						*categories				
1 Ventricle						1 Ventricle				
2 Thalamus						2 Thalamus				
3 Grey m.						3 Grey m.				
4 White m.						4 White m.				
*data						*data				
1 1.94289 0.193659 65024.56						1 -1.55126 0.544895 4093.528				
2 0.95265 -0.10159 28244.56						2 0.74859 0.051582 1452.452				
3 1.02428 0.007544 19205.73						3 1.365296 -1.235632 424.4826				
4 -5.85265 0.009442 42458.51						4 1.45528 0.214852 1843.452				
1 1.95215 0.194528 65025.36						1 -1.54865 0.548262 4094.624				
2 0.95492 -0.10236 28245.03						2 0.74823 0.052518 1452.687				
3 1.02579 0.007546 19205.85						3 1.364485 -1.24586 425.3852				
4 -5.85469 0.009452 42458.56						4 1.45821 0.212548 1845.325				
*end						*end				
* B11 report file [raw data analysis] <1/4/2016 9:13:30 PM>						* B11 report file [raw data analysis] <1/4/2016 8:44:05 PM>				
* Data file name: "3TA.sel"						* Data file name: "1.5tA.sel"				
* Selected features [3 out of 3]						* Selected features [3 out of 3]				
Kurtosis [#1/#1]; p.mean=-4.81832E-001, p.std= 3.34194E+000						Kurtosis [#1/#1]; p.mean= 5.05023E-001, p.std= 1.30135E+000				
Skewness [#2/#2]; p.mean= 2.72776E-002, p.std= 1.13693E-001						Skewness [#2/#2]; p.mean=-1.07104E-001, p.std= 7.25204E-001				
Mean [#3/#3]; p.mean= 3.87335E+004, p.std= 1.84891E+004						Mean [#3/#3]; p.mean= 1.95399E+003, p.std= 1.43242E+003				
Feature vector standardized: NO						Feature vector standardized: NO				
* Results [raw-data analysis]						* Results [raw-data analysis]				
> Fisher coefficient, F = 1000000.0						> Fisher coefficient, F = 1000000.0				
> 1-NN classification of raw data						> 1-NN classification of raw data				
Missclassified data vectors: 0/8 [or 0.00%]						Missclassified data vectors: 0/8 [or 0.00%]				
