# Supplementary material for: Parameter set for computer-assisted texture analysis of fetal brain
Source: BMC Res Notes. 2016 Nov 25;9:496. doi: 10.1186/s13104-016-2300-3 (PMC5124296; doi:10.1186/s13104-016-2300-3)
Supplement: Supplementary file 3 — Additional file 3: Dataset 3. Raw texture analysis/Fisher coefficient: ➤ appendix 1, ➤ appendix 2, ➤ appendix 3. [file 13104_2016_2300_MOESM3_ESM.zip › dataset 3_appendix2_Parameter set for Computer-Assisted Texture Analysis of Fetal Brain.pdf.pdf]

APPENDIX 2

Dataset 3: Raw texture Analysis / Fisher Coefficient

Column1 Column2 Column3 Column4 Column5	Column7 Column8 Column9 Column10 Column11 Column12
**Fig. 5 | 3T: T2 TSE - sample X vs Y**	**Fig. 6 | 3T: T2 TSE - sample X vs Y**
MaZda report	MaZda report
*features	*features
1 Kurtosis	1 Kurtosis
2 Skewness	2 Skewness
3 Mean	3 Mean
*categories	*categories
1 Ventricle	1 Ventricle
2 Thalamus	2 Thalamus
3 Grey m.	3 Grey m.
4 White m.	4 White m.
*data	*data
1 1 1.85041 -0.07609 65254.05	1 1 1.145853 0.254868 4016.325
2 2 5.58946 0.00582 31458.55	2 2 0.945288 0.59629 1542.36
3 3 4.84963 0.00546 20315.89	3 3 -1.54962 -0.54283 512.6458
4 4 -2.91345 0.27858 41046.56	4 4 -2.54869 1.154355 2541.659
5 1 1.95215 0.194528 65025.36	5 1 -1.54865 0.548262 4094.624
6 2 0.95492 -0.10236 28245.03	6 2 0.74823 0.052518 1452.687
7 3 1.02579 0.007546 19205.85	7 3 1.364485 -1.24586 425.3852
8 4 -5.85469 0.009452 42458.56	8 4 1.45821 0.212548 1845.325
*end	*end
* B11 report file [raw data analysis] <1/5/2016 4:56:19 PM>	* B11 report file [raw data analysis] <1/5/2016 1:53:31 PM>
* Data file name: "3TA.sel"	* Data file name: "1.5tA.sel"
* Selected features [3 out of 3]	* Selected features [3 out of 3]
Kurtosis [#1/#1]; p.mean= 9.31777E-001, p.std= 3.77548E+000	Kurtosis [#1/#1]; p.mean= 1.88825E-003, p.std= 1.60579E+000
Skewness [#2/#2]; p.mean= 4.03670E-002, p.std= 1.30323E-001	Skewness [#2/#2]; p.mean= 1.28769E-001, p.std= 7.38315E-001
Mean [#3/#3]; p.mean= 3.91262E+004, p.std= 1.81110E+004	Mean [#3/#3]; p.mean= 2.05388E+003, p.std= 1.41157E+003
Feature vector standardized: NO	Feature vector standardized: NO
* Results [raw-data analysis]	* Results [raw-data analysis]
> Fisher coefficient, F = 897.4	> Fisher coefficient, F = 144.1
> 1-NN classification of raw data	> 1-NN classification of raw data
Missclassified data vectors: 0/8 [or 0.00%]	Missclassified data vectors: 1/8 [or 12.50%]
	Sample No: 8; Category: 4; ClassResult: 2
