# Supplementary material for: Parameter set for computer-assisted texture analysis of fetal brain
Source: BMC Res Notes. 2016 Nov 25;9:496. doi: 10.1186/s13104-016-2300-3 (PMC5124296; doi:10.1186/s13104-016-2300-3)
Supplement: Supplementary file 3 — Additional file 3: Dataset 3. Raw texture analysis/Fisher coefficient: ➤ appendix 1, ➤ appendix 2, ➤ appendix 3. [file 13104_2016_2300_MOESM3_ESM.zip › dataset 3_appendix3_Parameter set for Computer-Assisted Texture Analysis of Fetal Brain.pdf.pdf]

APPENDIX 3

Dataset 3: Raw texture Analysis / Fisher Coefficient

Column1	Column2	Column3	Column4	Column5	Column6	Column7	Column8	Column9	Column10	Column11
*features										
1 Kurtosis						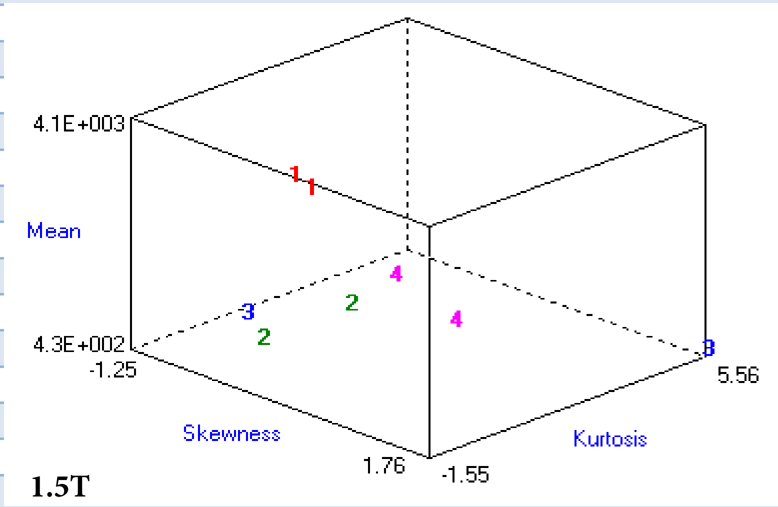				
2 Skewness										
3 Mean										
*categories										
1 Ventricle										
2 Thalamus										
3 Grey m.										
4 White m.										
*data										
1 1 -1.01357 0.177188 3954.658										
2 2 -1.13363 -0.09546 1245.949										
3 3 5.56318 1.755691 645.3288										
4 4 1.16023 0.940748 1624.583										
5 1 -1.54865 0.548262 4094.624										
6 2 0.74823 0.052518 1452.687										
7 3 1.364485 -1.24586 425.3852						**2 images from 2 different T2 sequences and different patients**				
8 4 1.45821 0.212548 1845.325										
*end										
* b11 report file [raw data analysis] <1/5/2016 5:39:42 PM>										
* Data file name: "1.5tA - T2-T2.sel"										
* Selected features [3 out of 3]										
Kurtosis [#1/#1]; p.mean= 8.24811E-001, p.std= 2.27129E+000										
Skewness [#2/#2]; p.mean= 2.93204E-001, p.std= 8.63623E-001										
Mean [#3/#3]; p.mean= 1.91107E+003, p.std= 1.38834E+003										
Feature vector standardized: NO										
* Results [raw-data analysis]										
> Fisher coefficient, **F = 448.6**										
> 1-NN classification of raw data										
Missclassified data vectors: 2/8 [or 25.00%]										
Sample No: 4; Category: 4; ClassResult: 2										
Sample No: 6; Category: 2; ClassResult: 4										
