# Supplementary material for: Parameter set for computer-assisted texture analysis of fetal brain
Source: BMC Res Notes. 2016 Nov 25;9:496. doi: 10.1186/s13104-016-2300-3 (PMC5124296; doi:10.1186/s13104-016-2300-3)
Supplement: Supplementary file 3 — Additional file 3: Dataset 3. Raw texture analysis/Fisher coefficient: ➤ appendix 1, ➤ appendix 2, ➤ appendix 3. [file 13104_2016_2300_MOESM3_ESM.zip › dataset 3_Parameter set for Computer-Assisted Texture Analysis of Fetal Brain.pdf]

Dataset 3: Raw texture Analysis / Fisher Coefficient: ➤ ➤appendix 1, ➤ ➤appendix 2, ➤ ➤ appendix 3.

1.5T

BEFORE
*label
16-bit Dicom
*features
1 I-dipersion
2 I-focus
3 Wavenhl_s-1
*categories
1 Ventricle
2 Thalamus
3 Grey m.
4 White m.
5 min
6 max
*data
5 0 -1 0
1 3.078876 0.092772 50325.31
2 47.93739 0.015724 5208.541
3 277.6537 -0.00045 113489.7
4 22.12487 0.031702 4132.11
6 307 1 120000
5 0 -1 0
1 2.723703 0.025749 48653.26
2 52.07033 0.000573 5329.235
3 305.8016 0.000172 97653.26
4 23.42985 0.078234 4025.37
6 307 1 120000
5 0 -1 0
1 3.125575 0.017607 51568.27
2 45.45708 0.002946 4912.589
3 306.8523 0.008627 105865.8
4 30.93632 0.117028 4125.368
6 307 1 120000
*end

AFTER
*label
8-bit BMP
*features
1 I-dipersion
2 I-focus
3 Wavenhl_s-1
*categories
1 Ventricle
2 Thalamus
3 Grey m.
4 White m.
5 min
6 max
*data
5 0 -1 0
1 0.945893 -1.03017 50120.65
2 2.551511 0.646327 5114.592
3 26.01978 -0.99997 113526.5
4 1.534392 -1.18804 4152.369
6 307 1 120000
5 0 -1 0
1 0.971307 0.98343 48529.33
2 2.546709 1.100645 5258.169
3 26.04218 -0.4804 98653.26
4 1.570257 0.460951 4020.326
6 307 1 120000
5 0 -1 0
1 0.978035 4.273099 51425.17
2 2.64966 0.47691 4952.368
3 26.59727 1.16 100758.6
4 1.46789 0.524722 4232.368
6 307 1 120000
*end

3T

*label
16-bit Dicom
*features
1 I-dipersion
2 I-focus
3 Wavenhl_s-1
*categories
1 Ventricle
2 Thalamus
3 Grey m.
4 White m.
5 min
6 max

*label
8-bit BMP
*features
1 I-dipersion
2 I-focus
3 Wavenhl_s-1
*categories
1 Ventricle
2 Thalamus
3 Grey m.
4 White m.
5 min
6 max

Dataset 3: Raw texture Analysis / Fisher Coefficient: ➤ ➤appendix 1, ➤ ➤appendix 2, ➤ ➤ appendix 3.

5 0 -1 0
1 17.20336 -0.00259 49715.15
2 1813.978 0.000548 5038.396
3 5387.888 0.004372 108903.8
4 674.609 0.001633 3873.634
6 5500 1 120000
5 0 -1 0
1 18.63379 0.002012 49352.54
2 1996.078 0.002892 5132.581
3 5482.43 -0.001 105846.3
4 694.3229 0.000963 3954.258
6 5500 1 120000
5 0 -1 0
1 16.82593 0.002841 50164.84
2 1971.235 0.012343 4985.364
3 5053.272 0.002426 98456.27
4 737.3618 0.002584 4385.369
6 5500 1 120000
*end

5 0 -1 0
1 0.988573 0.336988 49622.21
2 2.507098 0.442475 5153.365
3 26.75551 0.343338 108485.2
4 1.504155 1.509819 3959.216
6 30 1 120000
5 0 -1 0
1 0.977259 0.467965 49246.42
2 2.900196 0.233987 5036.859
3 26.56437 2.810099 105754.3
4 1.478277 0.367272 3956.368
6 30 1 120000
5 0 -1 0
1 0.96105 1.68165 50015.26
2 2.861014 -3.33799 4995.268
3 25.41159 -0.65439 99356.33
4 1.550517 7.456392 4458.369
6 30 1 120000
*end
