# Supplementary material for: Parameter set for computer-assisted texture analysis of fetal brain
Source: BMC Res Notes. 2016 Nov 25;9:496. doi: 10.1186/s13104-016-2300-3 (PMC5124296; doi:10.1186/s13104-016-2300-3)
Supplement: Supplementary file 4 — Additional file 4: Dataset 4. Data for supplemental trial runs: 36 × 1.5 T; 36 × 3 T; group 1: 20–28 weeks; group 2: 29–40 weeks. [file 13104_2016_2300_MOESM4_ESM.zip › dataset 4_Parameter set for Computer-Assisted Texture Analysis of Fetal Brain.pdf]

Dataset 4. Supplemental trial runs

Parameter set for Computer-Assisted Texture Analysis of Fetal Brain

		ROI	*Kurtosis*	*Skewness*	*Variance*	*Mean*	*I_{FOCUS}*	*$I_{DISPERSION}$*	*V_{ROI}*
GROUP 1	1.5T	Ventricle	1.145853	0.254868	10485.655	4016.325	0.222427	2.610759	
	Axial	Thalamus	0.945288	0.59629	75215.254	1542.36	0.630803	48.76635	1.86E+10
	PD	Grey m.	-1.54962	-0.54283	114254.78	512.6458	0.350297	222.8727	
	TRUFI	White m.	-2.54869	1.154355	51024.546	2541.659	-0.45292	20.0753	
					F (5,15)= 8.09 P= 0.0007				
	3T	Ventricle	1.85041	-0.07609	975812.25	65254.05	-0.04112	14.95405	
	Axial	Thalamus	5.58946	0.00582	58562405	31458.55	0.001041	1861.574	9.11E+15
	T2 TSE	Grey m.	4.84963	0.00546	78124874	20315.89	0.001126	3845.506	
		White m.	-2.91345	0.27858	26452160	41046.56	-0.09562	644.4428	
					F (5,15)= 5.77 P= 0.004				
	1.5T	Ventricle	-1.54865	0.548262	11254.655	4094.624	-0.35403	2.748642	
	Coronal	Thalamus	0.74823	0.052518	65345.655	1452.687	0.07019	44.98261	1.49E+10
	T2	Grey m.	1.364485	-1.24586	104578.36	425.3852	-0.91306	245.8439	
	HASTE	White m.	1.45821	0.212548	42546.896	1845.325	0.14576	23.05658	
					F (5,15)=7.88 P= 0.0008				
	3T	Ventricle	1.95215	0.194528	991233.43	65025.36	0.099648	15.2438	
	Coronal	Thalamus	0.95492	-0.10236	50135689	28245.03	-0.10719	1775.027	7.37E+15
	T2 TSE	Grey m.	1.02579	0.007546	71546872	19205.85	0.007356	3725.265	
		White m.	-5.85469	0.009452	25468245	42458.56	-0.00161	599.8377	
					F (5,15)= 5.9 P= 0.003				
	1.5T	Ventricle	-1.01357	0.177188	5452.6548	3954.658	-0.17482	1.378793	
	Sagittal	Thalamus	-1.13363	-0.09546	65348.235	1245.949	0.084207	52.44858	1.35E+10
	T2	Grey m.	5.56318	1.755691	98653.485	645.3288	0.315591	152.8732	
	HASTE	White m.	1.160233	0.940748	36542.852	1624.583	0.810827	22.49368	
					F (5,15)= 6.5 P= 0.002				
	3T	Ventricle	-2.96548	0.125462	901258.75	64258.54	-0.04231	14.02551	
	Sagittal	Thalamus	3.258757	-0.00542	44587548	22875.36	-0.00166	1949.152	6.83E+15
	T2 TSE	Grey m.	4.587545	0.021453	69854525	19854.87	0.004676	3518.256	
		White m.	1.954232	0.581245	28746290	48235.42	0.297429	595.9581	
					F (5,15)= 2.88 P= 0.003				
	1.5T	Ventricle	2.548562	0.32856	3542.6584	3598.365	0.12892	0.984519	
	Axial	Thalamus	0.958214	0.865341	70365.895	1125.649	0.903077	62.51141	1.81E+10
	T2	Grey m.	0.953482	0.728439	119548.03	652.3699	0.763977	183.2519	
	HASTE	White m.	-1.8553	0.289271	32015.699	1025.649	-0.15592	31.21508	
					F (5,15)= 4.94 P= 0.007				
	3T	Ventricle	2.015486	0.146252	912458.23	65346.29	0.072564	13.96343	
	Axial	Thalamus	5.854226	0.004821	58645212	33246.81	0.000824	1763.935	6.24E+15
	T2 TSE	Grey m.	1.042153	0.021356	75461580	21054.65	0.020492	3584.081	
		White m.	-4.86461	0.002987	30548548	49542.86	-0.00061	616.6085	
					F (5,15)= 6.21 P= 0.0003				
	1.5T	Ventricle	1.325483	0.365295	3954.6852	4026.659	0.275593	0.982126	
	Coronal	Thalamus	1.654895	-1.00358	80159.355	1258.621	-0.60643	63.68822	2.06E+10
	T2	Grey m.	-0.36428	0.123589	125486.03	519.3626	-0.33927	241.6155	
	HASTE	White m.	0.968753	-0.85846	30051.37	1128.059	-0.88615	26.63989	
					F (5,15)= 4.83 P= 0.008				
	3T	Ventricle	-1.72156	0.354625	898687.46	65169.28	-0.20599	13.79005	
	Coronal	Thalamus	3.954856	0.001254	46582452	37584.55	0.000317	1239.404	6.41E+15
	T2 TSE	Grey m.	-1.54863	-0.00545	66456982	20548.69	0.003521	3234.123	
		White m.	1.865426	0.005253	20128451	41548.09	0.002816	484.4615	
					F (5,15)= 5.4 P= 0.005				
GROUP 2	1.5T	Ventricle	0.985425	0.235859	8569.3255	3879.582	0.239347	2.208827	
	Sagittal	Thalamus	2.58462	-0.67483	68256.214	1154.687	-0.26109	59.11231	1.66E+10
	PD	Grey m.	-3.58427	-0.35886	114582.05	548.3298	0.100121	208.9656	
	TRUFI	White m.	1.657523	0.157988	29504.365	1058.695	0.095316	27.86861	
					F (5,15)= 5.48 P= 0.005				
	3T	Ventricle	1.025469	3.86E-06	1005425.9	65534.57	3.76E-06	15.34192	
	Sagittal	Thalamus	-5.01482	-0.00246	31548822	20458.71	0.00049	1542.073	6.06E+15
	T2 TSE	Grey m.	-2.98452	1.54E-06	71548587	18254.58	-5.2E-07	3919.487	
		White m.	0.985452	4.53E-04	25462085	42458.56	0.00046	599.6926	
					F (5,15)= 4.9 P= 0.007				
	1.5T	Ventricle	0.985428	2.13E-03	6895.3213	4078.659	0.002159	1.690585	
	Axial	Thalamus	1.548926	0.028586	70548.598	1254.852	0.018455	56.22063	1.92E+10
	PD	Grey m.	-0.98288	0.385869	124585.67	456.8958	-0.39259	272.6785	
	TRUFI	White m.	1.585756	0.085486	33548.287	1185.27	0.053909	28.30434	
					F (5,15)= 5.22 P= 0.006				
	3T	Ventricle	1.845822	0.025362	854628.85	61545.02	0.01374	13.88624	
	Axial	Thalamus	-3.55682	0.001546	59452528	30187.85	-0.00043	1969.419	8.09E+15
	T2 TSE	Grey m.	0.525468	0.012526	71548862	19845.64	0.023837	3605.269	
		White m.	-3.84525	0.052856	19857560	48623	-0.01374	408.3985	
					F (5,15)= 5.24 P= 0.006				
	1.5T	Ventricle	0.985483	-0.36285	5426.3286	4058.033	-0.36819	1.337182	
	Coronal	Thalamus	1.255886	-0.48822	84256.329	1354.852	-0.38875	62.18858	2.04E+10
	T2	Grey m.	2.548587	0.025585	122364.37	589.4522	0.010039	207.59	
	HASTE	White m.	1.548628	0.924486	29504.851	1205.254	0.596971	24.48019	
					F (5,15)= 5.11 P= 0.006				
	3T	Ventricle	1.048575	0.032385	966546.54	61464.65	0.030885	15.72524	
	Coronal	Thalamus	1.985456	-0.32823	58455282	37525.26	-0.16532	1557.758	7.79E+15
	T2 TSE	Grey m.	-2.84526	0.004256	70184496	19046.21	-0.0015	3684.959	
		White m.	1.022236	0.021528	19464664	47466.56	0.02106	410.0711	
					F (5,15)= 5.26 P= 0.005				
	1.5T	Ventricle	0.985424	0.589485	6895.3287	3958.159	0.598204	1.742055	
	Sagittal	Thalamus	0.845217	0.258786	73256.854	1456.852	0.306177	50.28433	1.58E+10
	T2	Grey m.	-1.24889	0.095858	105896.46	552.8468	-0.07676	191.5476	
	HASTE	White m.	-2.69853	0.954887	36854.269	1754.825	-0.35385	21.00168	
					F (5,15)= 6.52 P= 0.002				
	3T	Ventricle	-1.54854	-0.00643	1058215.5	65428.35	0.004151	16.17365	
	Sagittal	Thalamus	0.946383	0.224628	59825262	38653.54	0.237355	1547.73	8.61E+15
	T2 TSE	Grey m.	3.545218	-0.07282	73453582	21628.27	-0.02054	3396.184	
		White m.	-0.25464	-0.05462	26925462	43843.54	0.214513	614.1261	
					F (5,15)= 6.09 P= 0.003				

36 x 1.5T; 36 x 3T; group 1: 20-28 weeks; group 2: 29-40 weeks.
