# Supplementary material for: Parameter set for computer-assisted texture analysis of fetal brain
Source: BMC Res Notes. 2016 Nov 25;9:496. doi: 10.1186/s13104-016-2300-3 (PMC5124296; doi:10.1186/s13104-016-2300-3)
Supplement: Supplementary file 2 — Additional file 2: Dataset 2. Effects of pull-down conversion: 12/16-bits grayscale DICOM to 8-bit BMP (RGB-24). [file dataset 2_Parameter set for Computer-Assisted Texture Analysis of Fetal Brain.pdf › dataset 2_Parameter set for Computer-Assisted Texture Analysis of Fetal Brain.pdf]

Dataset 2: Effects of Pull-Down Conversion: 12/16-bits grayscale DICOM to 8-bit BMP (RGB-24).

EFFECTS OF PULL-DOWN CONVERSION

**A) WAVELETS**		Ventricle		Thalamus		Grey m.		White m.	
BEFORE	DICOM	3T	1.5T	3T	1.5T	3T	1.5T	3T	1.5T
	Allocated	16 bits	16 bits	16 bits	16 bits	16 bits	16 bits	16 bits	16 bits
	Usable	16 bits	12 bits	16 bits	12 bits	16 bits	12 bits	16 bits	12 bits
	SIZE	448x448	256x256	448x448	256x256	448x448	256x256	448x448	256x256
	WavEnHL	49715.15	50325.31	5038.396	5208.541	108903.8	113489.7	3873.634	4132.11
	WavEnHL	49352.54	48653.26	5132.581	5329.235	105846.3	97653.26	3954.258	4025.37
	WavEnHL	50164.84	51568.27	4985.364	4912.589	98456.27	105865.8	4385.369	4125.368
		P>0.05		P>0.05		P>0.05		P>0.05	
**B) WAVELETS**		Ventricle		Thalamus		Grey m.		White m.	
AFTER	BMP	3T	1.5T	3T	1.5T	3T	1.5T	3T	1.5T
	Allocated	8 bits	8 bits	8 bits	8 bits	8 bits	8 bits	8 bits	8 bits
	Usable	8 bits	8 bits	8 bits	8 bits	8 bits	8 bits	8 bits	8 bits
	SIZE	448x448	256x256	448x448	256x256	448x448	256x256	448x448	256x256
	WavEnHL	49622.21	50120.65	5153.365	5114.592	108485.2	113526.5	3959.216	4152.369
	WavEnHL	49246.42	48529.33	5036.859	5258.169	105754.3	98653.26	3956.368	4020.326
	WavEnHL	50015.26	51425.17	4995.268	4952.368	99356.33	100758.6	4458.369	4232.368
		P>0.05		P>0.05		P>0.05		P>0.05	
**C) HISTOGRAM**		Ventricle		Thalamus		Grey m.		White m.	
BEFORE	DICOM	3T	1.5T	3T	1.5T	3T	1.5T	3T	1.5T
	Kurtosis	-0.52357	0.286542	0.885496	0.345268	1.958462	-1.06483	0.948217	0.482569
	Skewness	0.001356	0.026583	0.000485	0.005429	0.008563	0.000483	0.001548	0.015298
	Variance	1125466	12368.33	58075263	74265.37	1.13E+08	143358.2	27204856	53452.89
	Mean	65421.33	4017.156	32015.42	1549.216	21056.9	516.32	40326.85	2415.964
	Ifocus	-0.00259	0.092772	0.000548	0.015724	0.004372	-0.00045	0.001633	0.031702
	Idispersion	17.20336	3.078876	1813.978	47.93739	5387.888	277.6537	674.609	22.12487
	Kurtosis	0.623549	0.126485	1.125868	0.231548	-2.12594	1.235947	0.568954	0.325775
	Skewness	0.001255	0.003257	0.003256	0.000133	0.002132	0.000212	0.000548	0.025487
	Variance	1215486	11235.36	60132568	75625.33	1.21E+08	152368.2	28645212	52369.45
	Mean	65230.24	4125.033	30125.37	1452.369	22135.24	498.2585	41256.33	2235.159
	Ifocus	0.002012	0.025749	0.002892	0.000573	-0.001	0.000172	0.000963	0.078234
	Idispersion	18.63379	2.723703	1996.078	52.07033	5482.43	305.8016	694.3229	23.42985
	Kurtosis	0.752648	0.131529	0.854623	0.256233	2.949322	0.948643	0.832013	0.549115
	Skewness	0.002138	0.002316	0.010548	0.000755	0.007156	0.008184	0.00215	0.064262
	Variance	1082359	12365.27	58321675	60152.36	98752135	153834.1	30125852	60189.27
	Mean	64326.85	3956.158	29586.37	1323.278	19542.22	501.3296	40856.27	1945.586
	Ifocus	0.002841	0.017607	0.012343	0.002946	0.002426	0.008627	0.002584	0.117028
	Idispersion	16.82593	3.125575	1971.235	45.45708	5053.272	306.8523	737.3618	30.93632
		P<0.05		P<0.05		P<0.05		P<0.05	
**D) HISTOGRAM**		Ventricle		Thalamus		Grey m.		White m.	
AFTER	BMP	3T	1.5T	3T	1.5T	3T	1.5T	3T	1.5T
	Kurtosis	-0.56279	-0.24888	0.426589	0.235894	0.36549	0.235849	-0.21585	0.534898
	Skewness	-0.18965	0.256385	0.188755	0.152465	0.125486	-0.23584	-0.32589	-0.63548
	Variance	171.1549	166.75	211.2355	210.1548	894.2649	865.3265	164.3268	166.2159
	Mean	173.1333	176.2884	84.25498	82.36485	33.42358	33.25648	109.2486	108.3269
	Ifocus	0.336988	-1.03017	0.442475	0.646327	0.343338	-0.99997	1.509819	-1.18804
	Idispersion	0.988573	0.945893	2.507098	2.551511	26.75551	26.01978	1.504155	1.534392
	Kurtosis	0.325849	0.23549	0.536268	0.231585	0.232159	0.326589	0.421658	0.45896
	Skewness	0.152486	0.231587	0.12548	0.254892	0.652389	-0.15689	0.154863	0.211558
	Variance	172.2466	170.2359	209.7584	204.3265	856.326	866.3159	164.127	165.2159
	Mean	176.2549	175.2649	72.3256	80.23158	32.23589	33.26587	111.0258	105.2158
	Ifocus	0.467965	0.98343	0.233987	1.100645	2.810099	-0.4804	0.367272	0.460951
	Idispersion	0.977259	0.971307	2.900196	2.546709	26.56437	26.04218	1.478277	1.570257
	Kurtosis	0.25896	-0.15326	0.15689	0.452628	-0.65188	0.365186	0.114287	0.426159
	Skewness	0.43548	-0.6549	-0.5237	0.215863	0.426583	0.423616	0.852168	0.223615
	Variance	170.259	172.3659	212.3358	215.3268	845.1537	855.2316	152.3158	158.1724
	Mean	177.1593	176.237	74.21698	81.26585	33.2586	32.15486	98.23548	107.7549
	Ifocus	1.68165	4.273099	-3.33799	0.47691	-0.65439	1.16	7.456392	0.524722
	Idispersion	0.96105	0.978035	2.861014	2.64966	25.41159	26.59727	1.550517	1.46789
		P>0.05		P>0.05		P>0.05		P>0.05	

Dataset 2 | Effects of Pull-Down Conversion: 3T vs 1.5T | a) wavelets: pre-degradation b) wavelets: post-degradation c) histogram: pre-degradation d) histogram: post-degradation. | WavEnHL values do not change significantly across the ROIs, which implies that this parameter is ideal for detection of anatomical or pathological fingerprints, regardless of the quality of the image. In terms of bits per pixel, histogram parameters vary significantly. At 16 bits, there is a difference between 3T and 1.5T; but there is no difference at 8 bits. Measurements depend on gestational age and technical settings of MR units. Sample descriptions: gestational age: 26 week+; brain plane: coronal; weighting: T-2; slice thickness: 5 mm; repetition time: 2075-2100; echo time: 80-85; imaging frequency: 125-130; pixel bandwidth: 450-500.
